# Ecological disruptive selection acting on quantitative loci can drive sympatric speciation

**DOI:** 10.1038/s41540-024-00332-w

**Published:** 2024-01-15

**Authors:** Pavithra Venkataraman, Supreet Saini

**Affiliations:** Department of Chemical Engineering, Indian Institute of Technology Bombay, Mumbai, 400 076 India

**Keywords:** Evolution, Systems biology

## Abstract

The process of speciation generates biodiversity. According to the null model of speciation, barriers between populations arise in allopatry, where, prior to biology, geography imposes barriers to gene flow. On the other hand, sympatric speciation requires that the process of speciation happen in the absence of a geographical barrier, where the members of the population have no spatial, temporal barriers. Several attempts have been made to theoretically identify the conditions in which speciation can occur in sympatry. However, these efforts suffer from several limitations. We propose a model for sympatric speciation based on adaptation for resource utilization. We use a genetics-based model to investigate the relative roles of prezygotic and postzygotic barriers, from the context of ecological disruptive selection, sexual selection, and genetic architecture, in causing and maintaining sympatric speciation. Our results show that sexual selection that acts on secondary sexual traits does not play any role in the process of speciation in sympatry and that assortative mating based on an ecologically relevant trait forces the population to show an adaptive response. We also demonstrate that understanding the genetic architecture of the trait under ecological selection is very important and that it is not required for the strength of ecological disruptive selection to be very high in order for speciation to occur in sympatry. Our results provide an insight into the kind of scenarios in which sympatric speciation can be demonstrated in the lab.

## Introduction

Understanding phenomenology and genetics of speciation is a longstanding problem in biology. Historically, allopatric speciation, which is driven by geographical isolation, has been considered the null model of speciation^1–3^. The likelihood of the occurrence of speciation in sympatry, where geography does not pose any hindrance to gene flow, was often thought to be an impossible phenomenon in nature^2,3^. However, several empirical evidences of sympatric speciation in nature have been found^4–21^. The study of sympatric speciation, however, has been limited because of the absence of a laboratory model. Therefore, identifying conditions and adjudging the likelihood of sympatric speciation is important to understanding forces that drive the generation of biodiversity.

Speciation is the “fission of a gene pool”^22^. To explain the cause of this “fission” and its maintenance in sympatry, several models have been proposed (reviewed systematically in ref. ^22,23^). These models, based on an idea first proposed by Maynard Smith^24^, describe disruptive selection acting on a trait of members of the population. In such a setting, an adaptive response of the population is to diverge into two groups.

Premating isolation in the form of preferential mating due to sexual selection (assortative mating) has been studied extensively^25–32^. The preference of mates can be based on the trait under ecological selection or selectively neutral marker traits. The genetic basis of assortativeness has been examined theoretically^33–38^.

Disruptive selection, also considered a driving force of speciation in sympatry, can be invoked to act on mating traits, secondary sexual characters, or physical traits that are ecologically relevant^24,25,39–51^.

These broad frameworks to decipher the conditions in which sympatric speciation may occur suffer from several limitations. First, disruptive selection, sexual or ecological, has been considered a major force that drives sympatric speciation^23^. The theory has shown that an extremely high strength of disruptive selection is needed for sympatric speciation to occur. However, examples from ecology where such stringent conditions may be met are not known^52,53^. Second, for a divergence to be maintained in a population, several models invoke sexual selection, where males invest in an ornament and females exhibit mating bias. These costs are not accounted for in models of sympatric speciation^54^. Third, assortative mating based on secondary sexual characters or neutral marker traits that have little ecological significance are used as traits on which disruptive sexual selection acts. The ecological relevance of such a framework is an open question^55^. Last, we do not yet know of the importance of genetic architecture in dictating the likelihood of sympatric speciation.

**Fig. 1:**
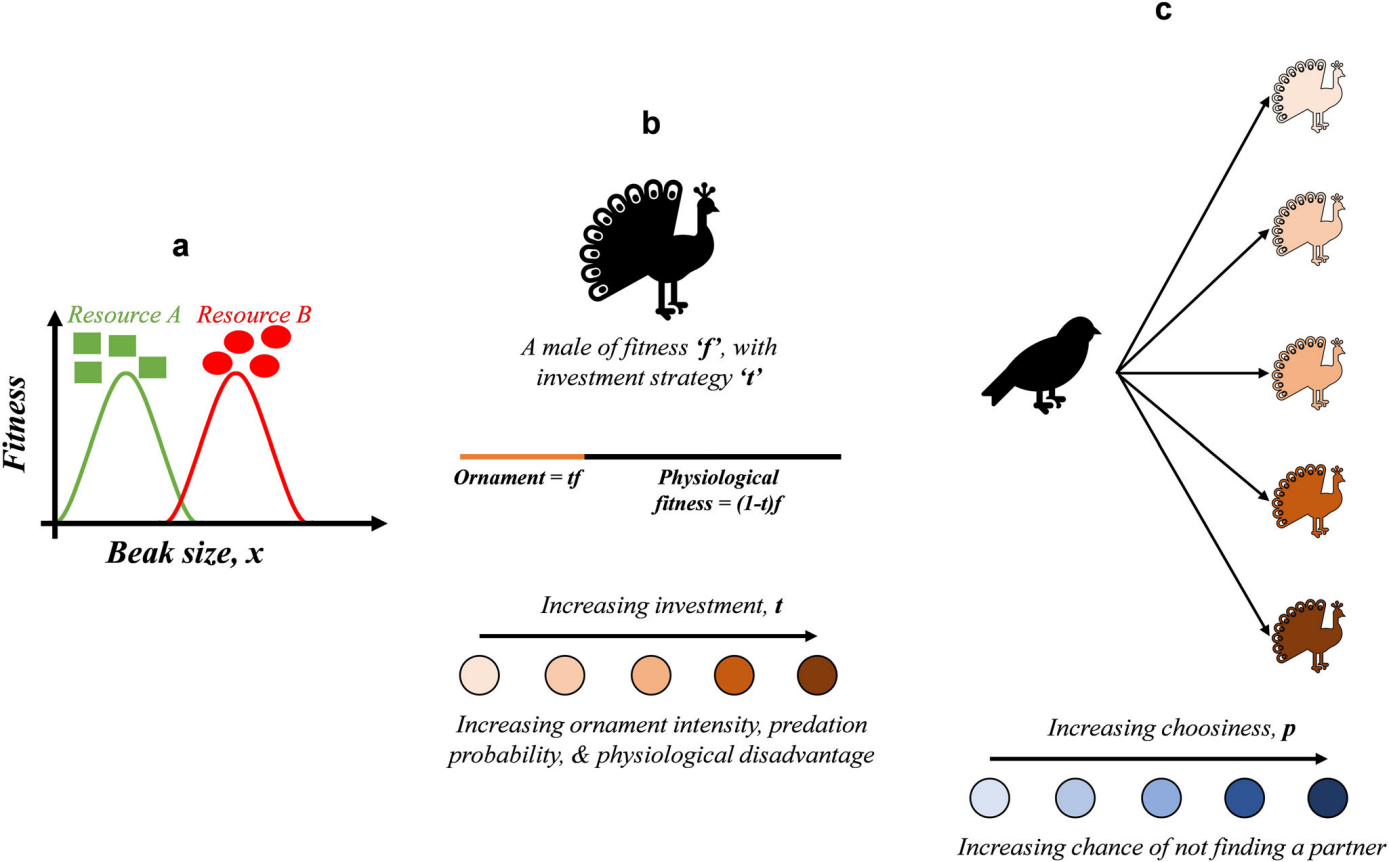
Model description. **a** The variation of fitness with beak size. In the hypothetical environment under consideration, there are two types of food resources, A and B. An individual with a small beak best utilizes resource A; while resource B is most effectively utilized with a larger beak. **b**, **c** Behavior of the males and females. In the bird population under consideration, the males invest a portion of their fitness to make an ornament, to attract the females. Increasing investment in making this ornament comes with costs, including exposure to predators and a physiological disadvantage. Females, on the other hand, are choosy. They first court the males in the population and then pick a mate based on his ornament intensity. A highly choosy female may not find a partner.

To address these limitations, we develop a multi-locus genetic model to investigate the likelihood of sympatric speciation in a (bird) population (Fig. 1). We invoke ecological disruptive selection on a physical trait (beak size), and sexual selection on female mating preference to investigate the relative roles of these two selection forces.

Our results show that assortative mating based on sexual selection does not play a role in creating or maintaining genetic divergence. We show how genetic divergence can be created when the strength of disruptive selection is low, thus enabling the understanding of sympatric speciation in nature. We also decipher the role of genetic architecture in driving sympatric speciation and provide a framework that can be used to empirically test sympatric speciation in the lab.

## Results

### Based on the strength of disruptive selection, three outcomes are possible

We start with a population where each individual has the same genotype for beak size, and hence the same beak size (=1). Similarly, every female has the same genotype for choosiness, and every male’s genotype for investment strategy is identical. The starting population’s females are not choosy (choosiness = 0), and the males do not make the ornament (investment strategy = 0). Each of the loci that control *x, p*, and *t* contribute equally to the phenotype.

In this setting, three responses are possible—(a) the entire population shifts to one of the niches (runaway selection), and (b) the population splitting into two (equally or unequally) and occupying both the niches, which we term as sympatric speciation. Alternatively, (c) we consider a population to be ill-adapted if, after 50 generations, individuals with a beak size equal to 1 exist.

**Fig. 2:**
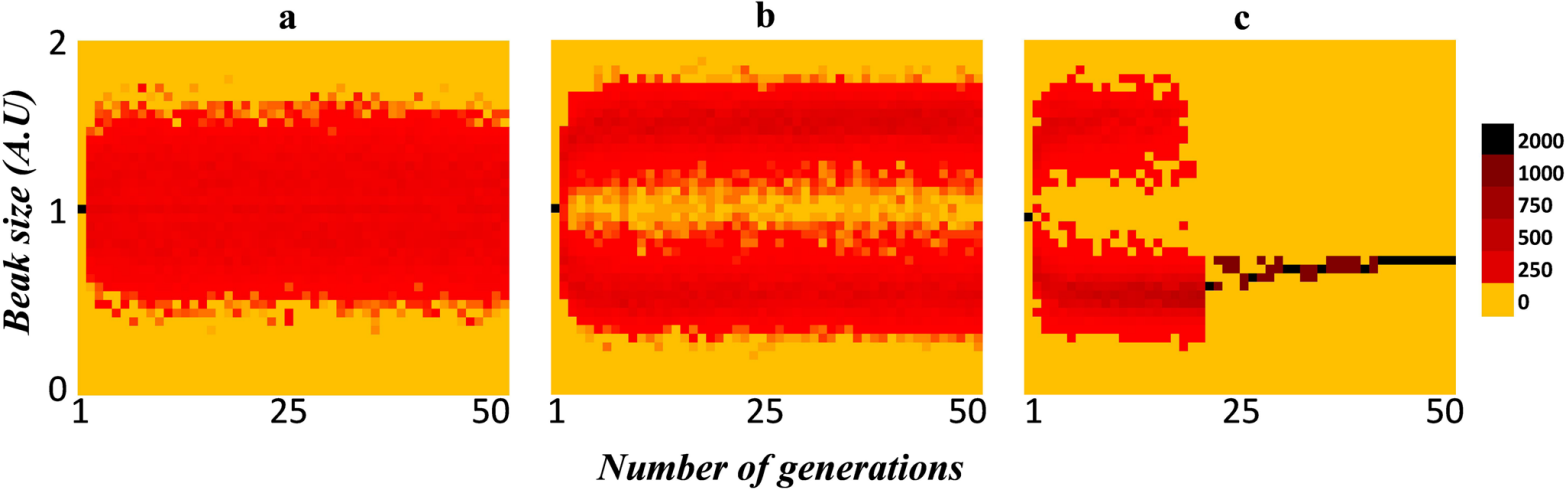
Possible evolutionary outcomes in this environment. In an environment where the strength of sexual selection is fixed (=5), the evolutionary trajectory of the trait relevant for fitness (beak size) is studied, by varying the strength of disruptive selection. When the strength of disruptive selection is **a** intermediate (=3.85), the population adapts to split into two distinct groups; **b** low (=1.667), the population does not split, and **c** high (=5), the population adapts to move to one of the two niches.

Figure 2 shows the three possible evolutionary trajectories of the population. In Fig. 2a, the population does not split into two groups. The scenario when the population splits into two distinct groups based on beak size is shown in Fig. 2b, and the possibility that the entire population shifts to one of the two niches is shown in Fig. 2c.

In Fig. 2c, the shift of the population to one of the niches occurs quite suddenly. It must be noted that just before the shift occurs, the frequency of well-adapted individuals in both niches is highly disproportionate. Therefore, for the population size to reach the desired size, it is possible that matings occur only between well-adapted individuals of this niche, resulting in the shift of the population to one of the two niches. In nature, such a shift of the population should be expected to occur more gradually, given that generations overlap.

### Likelihood and intensity of split depends on the strength of disruptive selection

In our framework, individuals with beak sizes 0, 1, and 2 have the lowest fitness. Other beak sizes confer fitness to the birds depending on the strength of disruptive selection. When disruptive selection is low, individuals acquire a significant gain in fitness without making a big change to beak size. But, as the strength of disruptive selection increases, small deviations from the starting beak size do not increase the fitness of the individual significantly. Thus, we hypothesize that an increase in selection pressure should lead to a greater chance of speciation in sympatry.

**Fig. 3:**
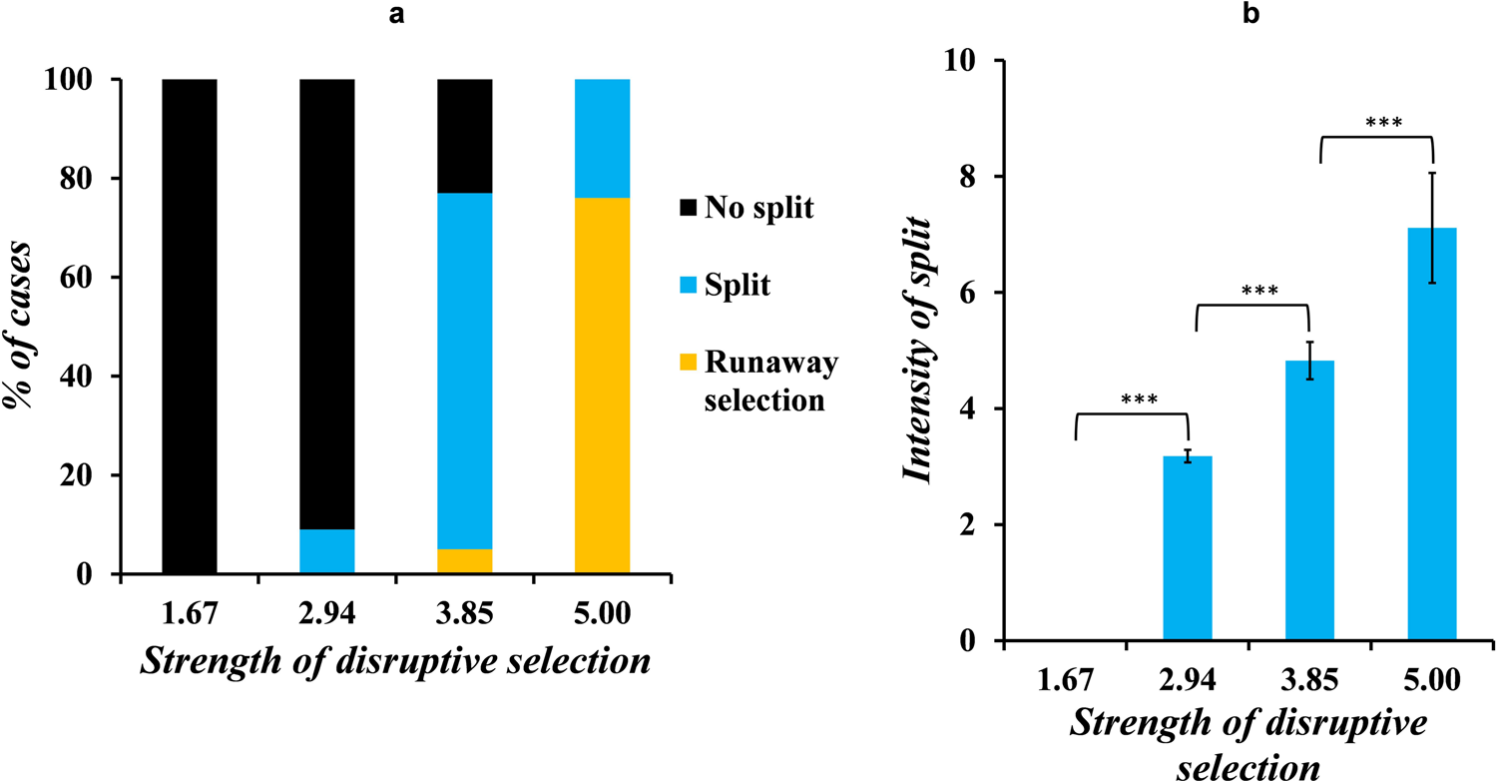
An increase in the strength of disruptive selection increases the tendency of the population to split, the intensity of split, and the likelihood of runaway selection. At low values of strength of disruptive selection, the population does not split into two. As the strength increases, the likelihood of the population splitting increases, along with the intensity, as shown in **a**, **b**. But, as depicted in **a**, when the strength of disruptive selection increases, the population’s tendency to undergo runaway selection also increases. We check if (i) the IoS (SoDS = 1.67) < IoS (SoDS = 2.94), (ii) if IoS (SoDS = 2.94) < IoS (SoDS = 3.85), and (iii) if IoS (SoDS = 3.85) < IoS (SoDS = 5), using one-tailed *t* tests. The *p* values (one-tailed *t* test) obtained for all three tests are less than 0.001. Since these *p* values (one-tailed *t* test) obtained indicate statistical reliability, we conclude that IoS (SoDS =1.67) < IoS (SoDS = 2.94) < IoS (SoDS = 3.85) < IoS (SoDS = 5). The error bars indicate means ± standard deviations.

For a given value of strength of disruptive selection, the population can exhibit any of the three responses, as shown in Fig. 2. However, the relative frequency of each response changes. Our results show that as the strength of disruptive selection increases, the population’s tendency to undergo a split increases, up to a certain extent (Fig. 3a). This is accompanied by an increase in the split intensity (quantified in equation 14) (Fig. 3b). For Fig. 3b, only cases where the population underwent a split are considered for calculation of the average split. So, for the case when strength of disruptive selection is ~1.67, there does not exist a value of the intensity of split. With a further increase in the strength of disruptive selection, the population increasingly responds to runaway selection.

Therefore, the intensity of split at high values of strength of disruptive selection drops as depicted in the heat plots in Supplementary Fig. 1. The effect of changing the strength of disruptive selection on beak size, choosiness of females, and investment strategy of the males, at the end of 50 generations, are shown as heat plots in Supplementary Figs. 2–4.

This non-monotonic behavior of the population as the strength of ecological disruptive selection increases can be explained by looking at the genetic architecture of the loci that control the beak size of the birds in the population. At low values of strengths of disruptive selection, even small deviations from that starting beak size (=1) are significantly beneficial. But, as the intensity of strength of disruptive selection increases, small deviations from the intermediate beak size are no longer as beneficial. In such a case, the population has to evolve beak sizes closer to either 0.5 or 1.5, thus reducing the width of the distribution of beaks centered around 0.5 and 1.5.

Increasing strength of disruptive selection makes the population’s trajectory increasingly dependent on the number of high-fitness males and females occupying a given niche in the first few generations. The number of such individuals being small, this dependence leads to an increase in probability of runaway selection as the strength of disruptive selection increases. Population dynamics obtained after simulations, when the strength of disruptive selection is ~2.94, ~3.85, and 5, are shown in Supplementary Fig. 5.

### Assortative mating based on sexual selection does not play any role in dictating the population behavior

Several models describe how divergence of mating preferences drives speciation in sympatry^23,25,55–57^. But, it has been argued that evolution and maintenance of divergence in isogenic populations is rare^23,55,58^. In our model, given that there is ecological disruptive selection acting on beak size, mating bias of females in the environment is likely to evolve based on a trait that is reflective of a male’s fitness^59^.

Sexual selection acts on the ornament size of the males and choosiness of the females. Since the type of ornament produced by males from both niches is the same, there is no divergence in mating preferences shown by the females. We check how female choosiness and male investment strategy vary with time in this modeling setting.

**Fig. 4:**
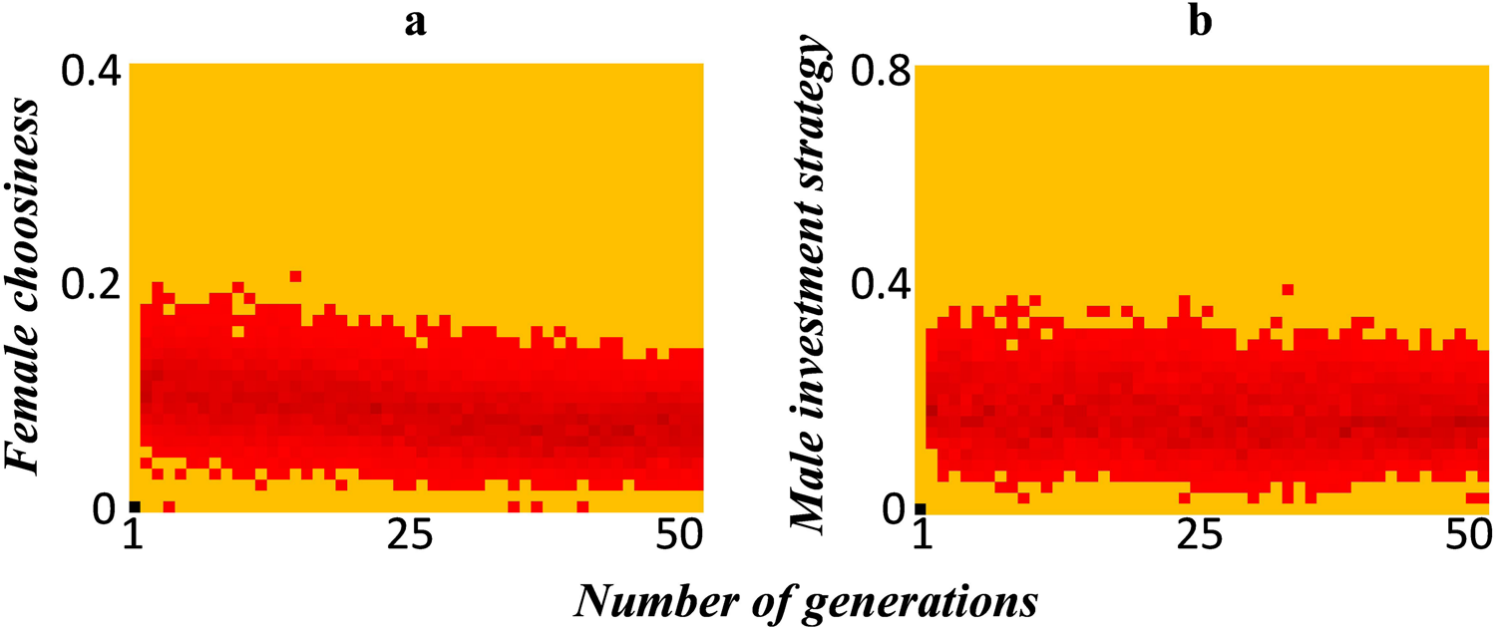
Temporal variation in the traits under sexual selection. In an environment in which the population splits into two distinct groups at the end of 50 generations, we show how **a** female choosiness, and **b** male investment strategy varies with time.

Figure 4a, b show the temporal change in female choosiness and male investment strategy, respectively, for the population that underwent a split at the end of 50 generations (shown in Fig. 2b). Supplementary Fig. 6 shows the temporal variation in these traits when the population does not split, or moves to one of the two niches. These traits that are under sexual selection appear to be evolving neutrally.

To test how the evolution of female choosiness and male investment changes if the nature of the starting population is changed, we perform simulations where the two traits are maximum (female choosiness = 0.4, male investment strategy = 0.8). All other parameters were kept the same as those in Fig. 2. As shown in Supplementary Fig. 7, a split in the population can occur at the end of 50 generations in this modeling setting. Supplementary Fig. 7 also shows the temporal variation of female choosiness and male investment strategy respectively.

Clearly, these two traits under sexual selection show no diversification. This leads us to investigate if sexual selection has any role to play at all, in dictating population dynamics in this modeling setting.

**Fig. 5:**
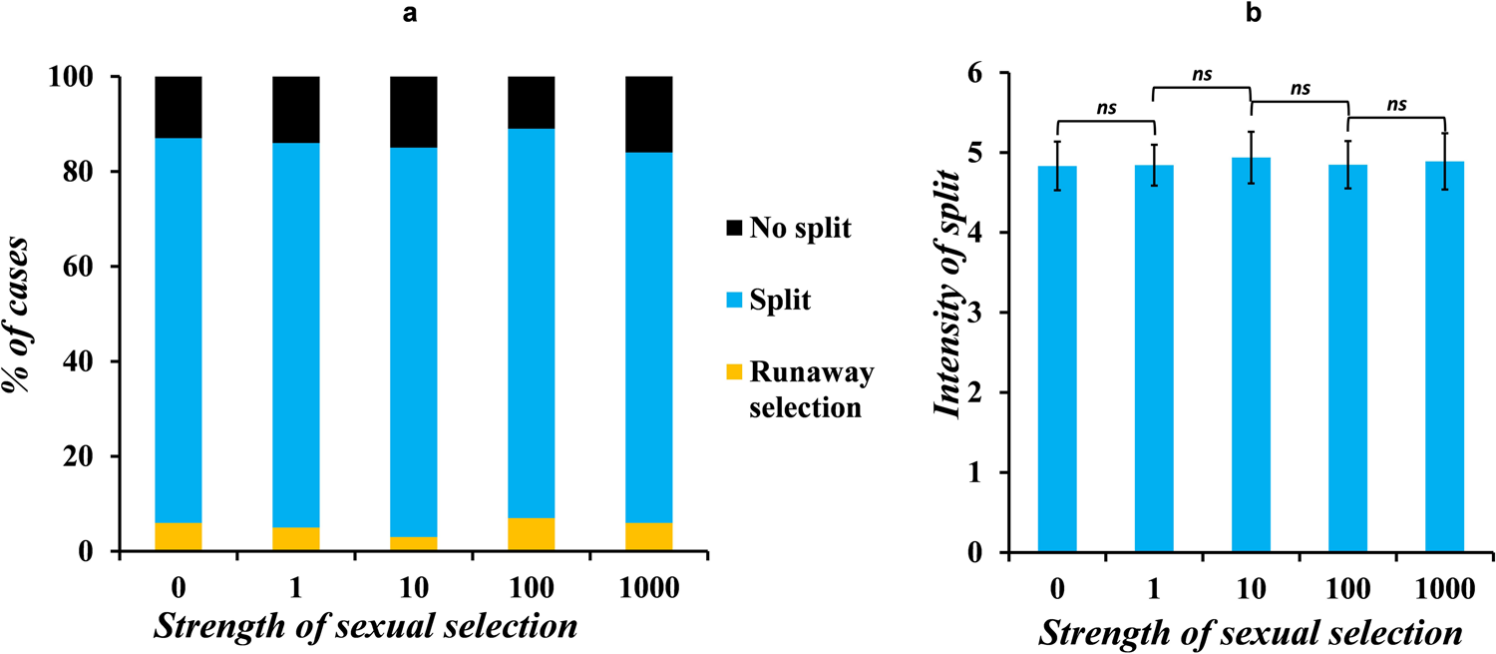
Sexual selection does not play a role in dictating the evolutionary trajectory of the population. In this modeling setting, sexual selection acts on two secondary sexual characters—choosiness of the females, and ornament size of the males. The figure describes the results obtained when the strength of disruptive selection is ~3.85. **a** The proportion of cases where the population undergoes a split, runaway selection, or does not split remains the same with increase in the magnitude of strength of sexual selection at play. **b** The intensity of split, whenever it occurs, also does not change with change in the strength of sexual selection. The error bars indicate means ± standard deviations.

We vary the strength of sexual selection, keeping the strength of disruptive selection fixed, to show that sexual selection does not play any role in causing or maintaining speciation in sympatry, as depicted in Fig. 5. See Supplementary Fig. 1 for data of split obtained in other cases. In fact, sexual selection may lead to a loss in genetic divergence by bringing together a choosy female from one niche and a male with an intense ornament from the other niche. The effect of changing the strength sexual selection, on beak size, choosiness of females, and investment strategy of the males, at the end of 50 generations are shown as heat plots in Supplementary Figs. 2–4.

**Fig. 6:**
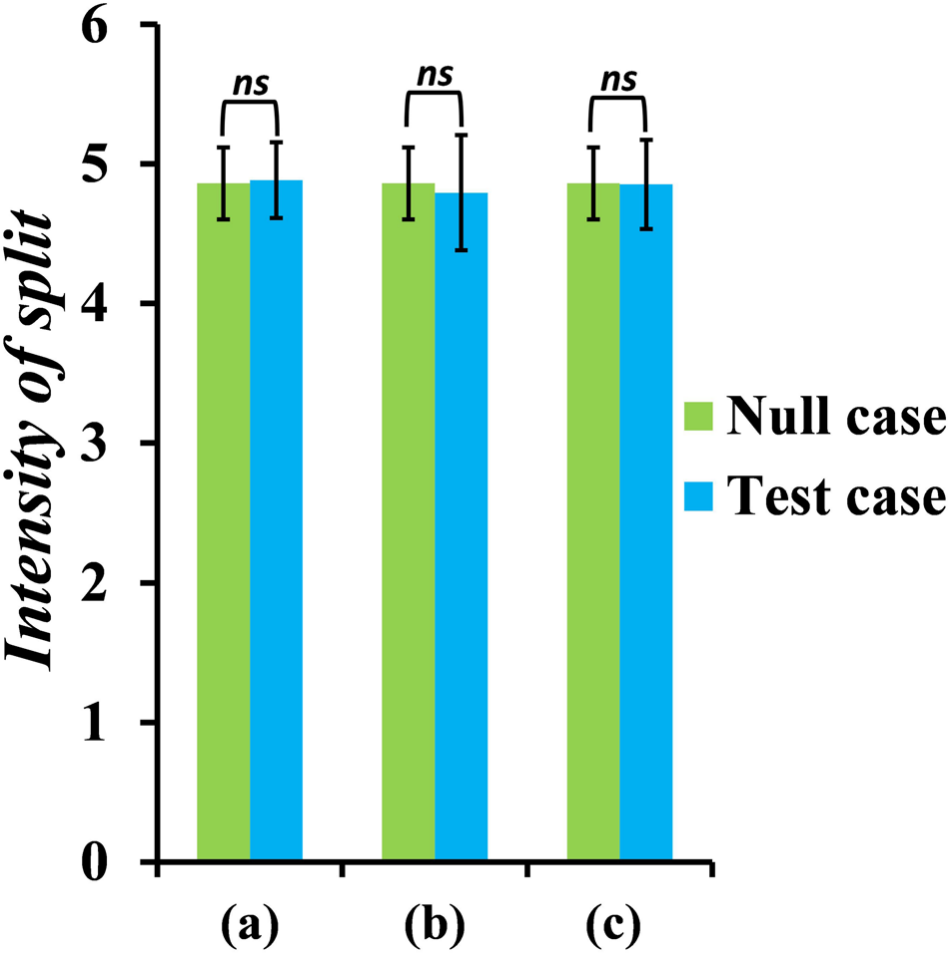
There is no effect on the intensity of split in the population when the secondary sexual traits of the females and males are absent. We check if the choosiness of the females or the investment strategy of the males has any role in dictating the evolutionary trajectory of the population. Null case here corresponds to the scenario where the females are choosy, and the males invest in making an ornament. The data shown here were obtained when the strength of disruptive selection and sexual selection were ~3.84 and 5, respectively. There is no change in the intensity of split in the population (*p* *=* ns, two-tailed *t* test), in the three test cases—**a** in an environment where the females are not choosy but the males invest in making an ornament, **b** when the males do not invest in making an ornament but the females are choosy, and **c** when the females are not choosy and the males do not make an ornament. The error bars indicate means ± standard deviations.

We next investigate the effect on the split intensity when (a) the females are not choosy, (b) when the males do not invest in making an ornament, and (c) when the females are not choosy, and the males do not invest in making an ornament. The results when the strength of disruptive selection is 3.85, and the strength of sexual selection is 5 are shown in Fig. 6.

When the females in the environment are not choosy, and/or the males in the environment do not invest in making an ornament, the intensity of the split in the population remains the same as that in the null case, where the females are choosy, and the males are making an ornament. This shows that sexual selection and the traits that it acts on have no role to play in the maintenance of genetic divergence in this setting.

### Introduction of dominance and unequal contribution individually and in combination in the loci controlling beak size reduces the likelihood of runaway selection

Complex traits are defined via unequal contributions from several loci^60–62^. Moreover, dominance relationships between alleles mean that the precise contribution from a single locus is dependent on the exact alleles present in an individual. We vary the genetic architecture of the loci that control evolvable traits in our model, and study the evolutionary effects of such modifications.

**Fig. 7:**
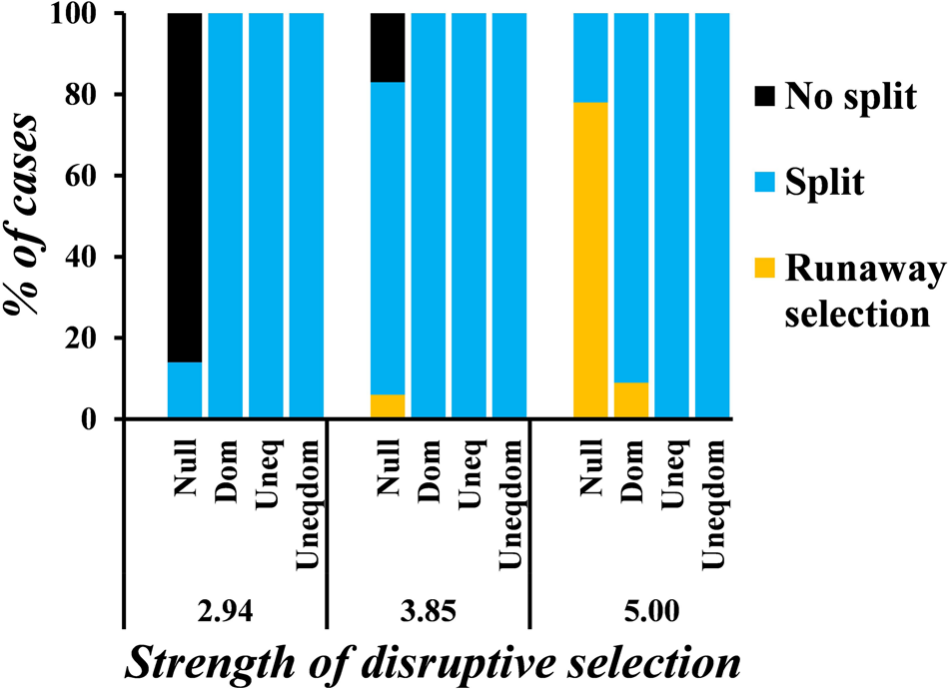
The likelihood of the population undergoing runaway selection decreases when there is a change in the genetic architecture. Results are shown for data obtained when the strength of sexual selection is 5. The graphs show the proportion of cases where the population does not split, undergoes runaway selection, or a split when the loci controlling beak size do not show dominance and contribute equally to the trait (*null*), when they show dominance relationships (*dom*), when they contribute unequally (*uneq*), and when they contribute unequally and have dominance relationships governing them (*uneqdom*).

Specifically, we make alterations to the genetic architecture by introducing (a) dominance, (b) unequal contributions, and (c) unequal contributions and dominance in the loci that control beak size. Our results, as depicted in Fig. 7, show that the likelihood of runaway selection decreases in all three cases. Beak size and intensity of split do not change with an increase in strength of sexual selection when the nature of loci controlling beak size is altered (Supplementary Figs. 8–15).

In the case where dominance relationships exist, the total number of possible beak sizes is lesser than the null case, but a step change in the genetic makeup confers twice the change in the beak size (in the no dominance case, an allele going from 0 to 1 increases the beak size by 0.05, but in the dominance case, it increases it by 0.1). This eases the possibility of the population splitting, by creating fit individuals in both niches, in lesser time. Hence, the likelihood of the population exhibiting runaway selection reduces.

In the case where the loci contribute unequally with or without dominance relationships, the number of accessible beak sizes increases, when compared to the null case where the loci contribute equally and show no dominance (since contributions of the loci are drawn from an exponential distribution, beak sizes like 0.59989 may be possible, compared to when they had to be multiples of 0.05 in the null case). As a result, creation of fit individuals in both niches is easier, and as a result, the likelihood of the population to split into two increases.

Both dominance relationships and unequally contributing loci enhance the possibility of acquiring beak sizes that enable survival in the presence of ecological disruptive selection.

**Fig. 8:**
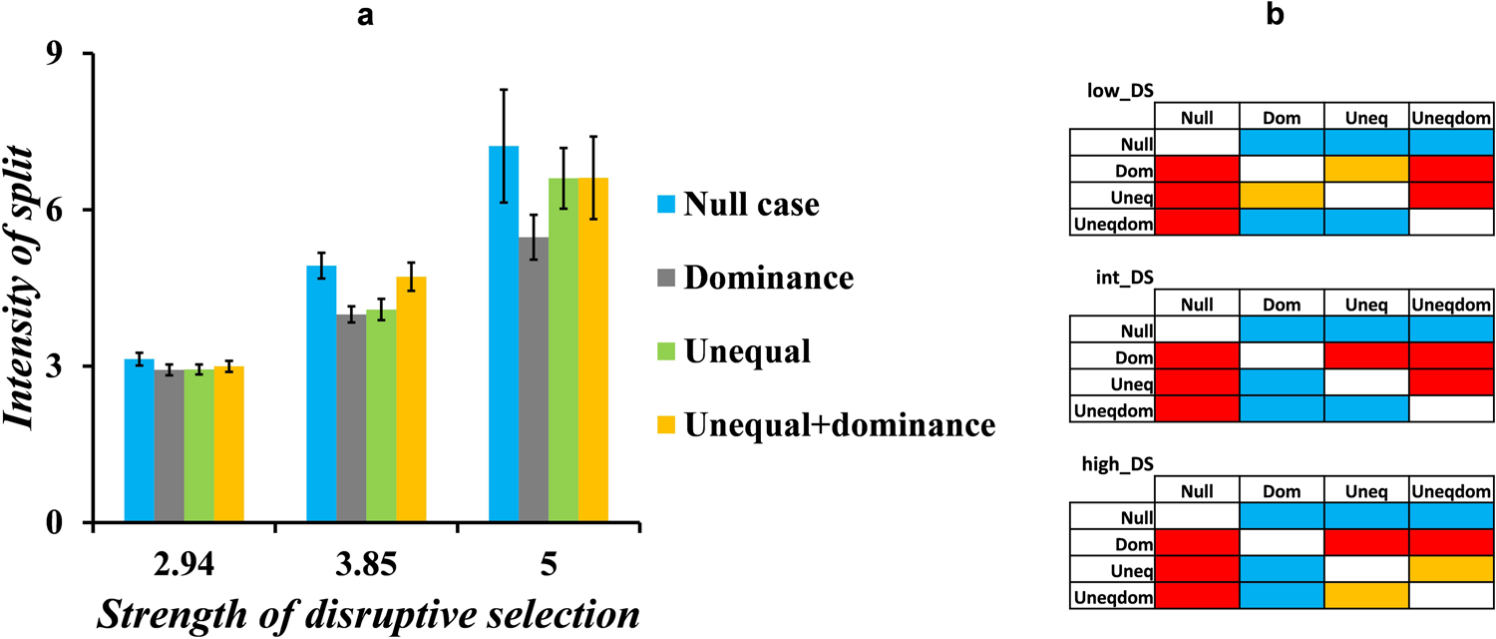
Intensity of split varies with variation in the genetic architecture. Varying the genetic architecture of the loci controlling beak size alters the intensity of split of the population. **a** shows the intensities of split at different values of strengths of disruptive selection. **b** is a table that is drawn after doing pairwise comparisons using Welch *t* test. A block is blue if IoS (row condition) >IoS (column condition), red block if IoS (row condition) <IoS (column condition), and yellow if IoS (row condition) = IoS (column condition). *low_DS*, *int_DS*, and *high_DS* correspond to the low, intermediate, and high strengths of disruptive selection, respectively. The error bars indicate means ± standard deviations.

We next check how the intensity of the split varies when changes are made to the genetic architecture of the loci controlling beak size. As shown in Fig. 8a, the intensity of split is the maximum in the null case (when there is no dominance in equally contributing loci), for all values of strength of disruptive selection. Figure 8b is a comparison of the intensities of split in the four different conditions of genetic architecture. These results are obtained by comparing the intensities of split using a Welch *t* test. All the *p* values obtained (less than 0.001, two-tailed *t* test) indicated the statistical reliability of the results.

Our results demonstrate that the intensity of split is the least when only dominance relationships in the loci that control beak size exist. Since the number of accessible beak sizes is the least in “dominance” condition, the mean beak sizes of the split populations are strictly 0.5 and 1.5. However, that is not the case with the other three conditions of genetic architecture. Therefore, when intensity of split is calculated according to Equation 14, the numerator is the least for the “dominance” condition.

## Discussion

What causes speciation in sympatry? The environmental conditions in which it occurs and the link of genetics with the likelihood of speciation in sympatry is not well understood. Several models have been able to demonstrate sympatric speciation in theory, by making a number of assumptions that often cannot be explained using first principles in biology^22,23,34,52–54,63–65^. For instance, disruptive selection acting on an ecologically irrelevant trait is unlikely to drive divergence in a trait relevant for fitness in an environment, which is necessary for speciation to occur in sympatry. In several models, assortative mating based on preferences is used to demonstrate sympatric speciation (reviewed exhaustively in ref. ^22^). However, these preferences come with costs, that are often not accounted for^25^. Also, the relative roles of the forces that cause speciation in sympatry, and genetic architecture of traits under selection are not very clearly understood.

Addressing these concerns, our model exhibits speciation in sympatry and explains the contributing forces and their roles in dictating the phenomenon. Since the starting population is isogenic for all the evolvable traits, it is unlikely that a divergence in mating preference based on secondary sexual traits, in order to survive the ecological disruptive selection will arise, at least until the population has undergone some genetic divergence in beak size^59^. In the absence of such a divergence in mating preference that constitutes a pre-zygotic barrier, we demonstrate that a split in the population can arise and be maintained with the help of postzygotic barriers that exist due to the ecological selection pressure.

We then investigate the role of strength of disruptive selection in dictating the evolutionary outcome of the population. Our results show that as strength of disruptive selection acting on the population increases, its tendency to show one of the two adaptive responses—split, or runaway selection—increases. An increase in disruptive selection increases the tendency of the population to split initially, but thereafter, the population is more likely to undergo runaway selection. This is despite accounting for competition within a niche. However, the tendency to exhibit a runaway response is greatly reduced when we include dominance and unequal contribution by loci in the genetics controlling the trait.

A common critique associated with demonstration of sympatric speciation is that it requires extremely strong disruptive selection (which is uncommon in nature). However, we demonstrate that for quantitative traits, split in the population can occur and be maintained for low disruptive selection values too. Hence, the proposition that genotypic divergence can occur when ecological disruptive selection is acting on a continuous quantitative trait controlled by quantitative trait loci is not unreasonable. This result not only provides an insight into the type of experiments that can be designed to demonstrate sympatric speciation, but also shows the importance of understanding the genetic architecture of the loci that control the trait under disruptive selection.

As discussed before, there are problems associated with modeling sexual selection based on traits that are not an indicator of an organism’s quality of genotype^66,67^. We address this issue by invoking sexual selection to act on a trait—an ornament—whose intensity is dependent on the organism’s fitness. We also account for the costs that come with producing such an ornament. Similarly, the females in the population are allowed to evolve choosiness based on this ornament. Sexual selection acts on these two traits such that it helps in producing a fit offspring, by increasing the probability of mating between fit males and females. We report that in the absence of a divergence in the marker traits produced by the males, sexual selection seems to have no role in dictating the population’s evolutionary fate. This result contradicts^41^ that assortative mating based on neutral marker traits can lead to a split in the population, and does not agree with a result reported previously^59^ that divergence in mating preferences is not required for the population to speciate in sympatry.

In fact, our results show that sexual selection in the environment tends to homogenize the genetic diversity in the population by favoring the mating of a very choosy female and a male with a bright ornament from different niches. A hybrid produced by this mating faces postzygotic barriers because of its low fitness and does not survive. The following strategies can prevent these “futile” cross-niche matings—(a) the individuals evolve niche-specific ornaments, and (b) the individuals mate assortatively based on the trait that is under ecological disruptive selection (beak size in this case). Exhibiting ornaments come with two costs for the male—increased risk of predation, and lower fitness left to perform physiological activities.

The nature of pre-zygotic barriers that will arise in this population to prevent cross-niche matings remains to be clearly ascertained. Will novel mutations allow for divergence of the ornament in the two niches? Also, how will a strategy that poses a pre-zygotic barrier evolve to get linked with the trait under ecological disruptive selection?

Although the choosiness and assortativeness of the females, and the investment strategy of the males of the starting population is zero, the entire population is not isogenic for these two traits (females and males differ in the loci that control choosiness, assortativeness, and investment strategy). This quickens the process of evolution of these traits. If we were to start with a population that was isogenic for all these traits, and if evolution were to occur only via mutations, the population would take much longer to attain the values of these phenotypes that we report.

Although sympatric speciation has been thought of an important route via which species diversity can arise, it has not been demonstrated experimentally. Our results provide an insight into an experimental design that can be adopted to demonstrate speciation in sympatry. Accordingly, we propose an experimental setup in which disruptive selection acts on a trait that is essential for metabolism, and hence survival. This trait should, favorably, be, controlled by several loci that contribute unequally. The strength of disruptive selection in such an experimental setup should be intermediate. Importantly, we show through our simulations that traits on which sexual selection acts in the presence of ecological disruptive selection need not be considered while designing experiments to demonstrate sympatric speciation.

## Methods

### Model description

Imagine a bird population, where the fitness of an individual depends on its *beak size, x*. The environment has two food resources (A corresponds to niche 1 and B to niche 2). The variation of fitness with beak size is shown in Fig. 1a.

The strength of ecological disruptive selection in this environment is quantified by Eq. 1.

1 $${Strength}\,{of}\,{disruptive}\,{selection}\,\left({SoDS}\right)=\frac{{{\rm{\mu }}}_{1}-{{\rm{\mu }}}_{2}}{{\sigma }_{1}+{\sigma }_{2}}$$

where ***μ***_*i*_ and ***σ***_***i***_ are the mean and standard deviation of the fitness distribution in ***i***th niche.

Males in the environment are capable of investing a portion of their fitness in making an ornament to attract the females. The intensity of this ornament is given by Eq. 2.

2 $${\rm{Ornament}}\,{\rm{intensity}}={\rm{male}}\,{\rm{investment}}\,{\rm{strategy}}* {\rm{fitness}}\,{\rm{of}}\,{\rm{the}}\,{\rm{male}}$$

However, males also incur costs for producing on ornament due to increased predation and by reducing their physiological fitness (Fig. 1b). Females, on the other hand, are capable of evolving “choosiness”, by virtue of which they preferentially pick mating partners based on ornament intensity. The ornament intensity is used by the females as a proxy of the fitness of the male it courts. A highly choosy female tends to pick a male with a high-intensity ornament for mating. But such a female risks not finding a partner (Fig. 1c), because—(a) a male with high-intensity ornament may not exist, or (b) the fitness of a high-intensity ornament male could be very low. The females in the environment meet males in the environment, and the likelihood of this meeting is dependent on the physiological fitness of the males.

### Modeling sexual selection

Apart from ecological disruptive selection, the role of sexual selection in causing a prezygotic barrier via premating isolation mechanisms is considered important^68–70^. In this environment, we invoke sexual selection^71^. It acts to drive assortative mating by enforcing a preference rule, based on a mating signal and a female’s preference for an elaborate mating signal. Such preference-based mating rules are common in sexual selection theory^72–74^.

We invoke sexual selection to act on two traits—ornament intensity of the males and the choosiness of the females. Both these traits are controlled by multiple loci.

### Modeling genetic architecture of the evolvable traits

#### Beak size

The starting isogenic population has a uniform beak size, ***x***, of 1, and is ill-adapted to utilize either of the two resources (see Fig. 1a). The individuals in the population are diploids and reproduce sexually. Beak size is genetically determined and is controlled by ***n***_***x***_ number of unlinked loci. A locus *i* contributes of ***δ***_***i***_ towards the beak size.

#### Male investment

Males spend a fraction of their fitness to make the ornament. This fraction is called the *investment strategy*, ***t***, and is a genetically determined phenotype. A total of ***n***_***t***_ number of loci control the investment strategy. The *i*th locus contributes of ***δ***_***i***_ towards the investment strategy.

#### Female choosiness

A female’s mate choice is biased, based on the intensity of the ornament exhibited by a male. This *choosiness*, ***p***, is a genetically determined phenotype that is controlled by ***n***_***p***_ number of unlinked loci. The *i*th locus contributes ***δ***_***i***_ towards determining the female’s choosiness.

All the loci in the population have only one of two alleles, ‘0’ or ‘1’. The value of a phenotype (***x***, ***p***, ***t***) is as shown in Eq. 3.

3 $${Value}\,{of}\,{phenotype}=\sum {\delta }_{j}{a}_{j}$$

where *δ*_*j*_ is the contribution of each allele towards a phenotype and ***a***_***j***_ is the numerical value (0 or 1) of the allele present at ***j***th locus.

Therefore, the lowest value of a phenotype is 0 and the highest is 2*n_loci_*δ, where ***n***_**loci**_ is the number of loci controlling the phenotype, ***δ*** is the contribution of each locus to the phenotype in the equal contribution case.

Investing in making an ornament comes with costs to the male. First, the fitness available for growth and reproduction decreases as investment towards making the ornament increases. If ***t*** is the fraction a male with fitness ***f***_***m***_ invests in making the ornament, the fitness available for physiological activities is shown in Eq. 4.

4 $${Physiological}\,{fitness}={f}_{m}* \left(1-t\right)* \left(1-\frac{{p}_{i}}{N}\,\right)$$

where, ***p***_***i***_ is the number of individuals in the niche that the male occupies and ***N*** is the population size.

Second, the probability that the male is predated upon increases as the ornament intensity increases. The probability ***p***_***e*s**_ is given by Eq. 5.

5 $${P\left(a\,{male}\,{escapes}\,{predation}\right),{p}_{{es}}\propto {\rm{e}}}^{\frac{-t}{1-t}}\,$$

where ***t*** is the investment strategy.

The females in the population pay a price for being choosy. As choosiness increases, the chance that a female finds a partner decreases. The probability that a female finds a mating partner is given by Eq. 6.

6 $$P\left(a\,{female}\,{finding}\,{partner}\right)\propto {{\rm{e}}}^{\frac{-8{p}^{3}}{{p}_{\max }^{3}}}* {f}_{f}* \left(1-\frac{{p}_{i}}{N}\right)$$

where, ***p*** is the choosiness, ***p***_**max**_ is the maximum choosiness genetically feasible and ***f***_***f***_ is the fitness of the female, ***p***_***i***_ is the number of individuals in the niche that the female occupies, and ***N*** is the population size.

In this setting, the ability of a bird to utilize a food resource also depends on the competition it faces. The environment is such that if all the birds evolve either small or large beaks (and utilize only one of the food sources), their fitness decrease due to increased competition. The value of fitness of a bird of beak size ***x*** in the niche ***i*** is given by Eq. 7.

7 $${fitness}={{\rm{e}}}^{\frac{-{\left(x-{\mu }_{i}\right)}^{2}}{2{\sigma }^{2}}}$$

where ***μ***_***i***_ is the beak size of the fittest individual in niche ***i***, ***σ*** is the standard deviation of the distribution of fitness with beak size. ***n***_***i***_ (*i* = 1,2) is the number of birds in niche ***i***, and ***N*** is the total number of birds in the population.

A female “meets” all the males in the population with a probability. The value of the probability is shown in Eq. 8.

8 $$P(a\,{meeting}\,{event}\,{between}\,a\,{male}\,{and}\,a\,{female})\propto {\left(1-t\right)* f}_{m}* \left(1-\frac{{p}_{i}}{N}\right)* {p}_{{es}}$$

where ***t*** is the investment strategy of a male, ***f***_***m***_ is his fitness, and ***p***_***es***_ is the probability with which he escapes predation.

One of these “meeting” events leads to a “mating” event, the probability of which is explained by Eq. 9.

9 $$P\left(a\,{mating}\,{event}\,{between}\,a\,{male}\,{and}\,a\,{female}\right)\propto {e}^{\alpha {pt}{f}_{m}}$$

where, ***α*** is the strength of sexual selection in the environment, ***p*** is the choosiness of the female, and ***tf***_***m***_ is the intensity of the ornament shown by the male.

One mating event leads to the production of one bird. Gender is assigned randomly. The generations are non-overlapping.

### Modeling of alterations in genetic architecture

*Dominance*—In the no-dominance case, we model the contributions of the loci controlling a phenotype according to the following rules:

- Each of the two alleles at a given locus can either be ‘0’ or ‘1’.
- The contribution of each allele to the phenotype, when there is ***n***_**loci**_ number of unlinked loci governing it, is given by Eq. 10.
  10 $${Contribution}\,{of}\,{each}\,{allele}\,{to}\,{phenotype},\delta =\frac{{maximum}\,{permissible}\,{value}\,{of}\,{the}\,{phenotype}}{2* {n}_{{\boldsymbol{loci}}}}$$
- The value of the phenotype is calculated as shown in Eq. 3.
  In the dominance case
- Allele ‘1’ is considered to be dominant over allele ‘0’.
- So, we consider the contribution of a locus and not an allele (because one allele masks the presence of another), and is calculated as shown in Eq. 11.
  11 $${Contribution}\,{of}\,{each}\,{locus}\,{to}\,{the}\,{phenotype},\Delta =2* \delta$$
  The contribution is modeled in this fashion to ensure that the maximum permissible beak size remains 2.
- If the number of loci with at least one ‘1’ is ${\boldsymbol{\gamma }},$ value of the phenotype is calculated as:

12 $${Value}\,{of}\,{the}\,{phenotype}=\gamma * \Delta$$

#### Unequal contribution

Quantitative traits are controlled by several loci that contribute small unequal values to the trait, and a few loci that contribute majorly to the value of the trait^60,61,75^. We model the unequal contribution of the loci that control three of the four evolvable traits—choosiness of the females, the investment strategy of the males, and the beak sizes of individuals in the population—by drawing random numbers from an exponential distribution of a set mean, and normalize these values such that the minimum and maximum beak sizes genetically accessible are 0 and 2, respectively. The mean of the exponential distributions is equal to the contribution of each of the loci if they were contributing equally.

#### Unequal contribution and dominance

We also model a case where the loci that control a trait show dominance relationships and contribute unequally to the quantitative trait. In this case, we draw the values of the contribution of each locus from an exponential distribution whose mean is twice the value of the contribution of these loci when they were contributing equally to the phenotype, without showing dominance relationships. These values are then normalized to ensure that the minimum and maximum beak sizes genetically accessible are 0 and 2, respectively.

### Statistics

The error bars in the plots shown correspond to standard deviation, unless specified otherwise. A comparison of two means was done using a two-tailed *t* test. A one-tailed *t* test was used to ascertain if one mean is greater than or lesser than another, and the value of *d* used in these tests was 0.001. In all cases, the significance level was 0.05.

*p* values >0.05, <0.05, <0.01, and <0.001 are indicated by **ns,** *, **, and *** respectively.

### Assumptions

- Only two alleles exist in the population at a given locus. One of the alleles is passed to the next generation with probability 0.5.
- Mutations between these two alleles occur at a fixed rate.
- All loci associated with the three straits are unlinked.

### Simulation steps

1. A female is chosen at random from the population, based on her choosiness and fitness.
2. The chosen female “meets” all the males in the population. The meeting probability is dependent only on the male’s fitness, as shown in Eq. 9.
3. A uniform random number is generated, and is used to pick one meeting event using Gillespie algorithm. The mating probability of the selected pair is calculated as given in Eq. 10. Another uniform random number is generated to decide if the mating event is successful or not, using Gillespie algorithm.
  Should a mating event take place, an offspring is born, and the population size increases by 1. The above steps are repeated until a fixed population size is reached. The first half of the individuals generated are females, while the second half are males.
4. Based on the beak sizes of individuals chosen for mating, the niche they occupy is decided. In a given niche, the frequency of different beak sizes is obtained, and normal distribution is fitted with this data. Split in the population is quantified as shown in Eq. 13.

13 $${Intensity}\,{of}\,{split}({IoS})=\frac{{\lambda }_{1}-{\lambda }_{2}}{{\chi }_{1}+{\chi }_{2}}$$

where ***λ***_***i***_ and ***χ***_***i***_ are the mean and standard deviation of the frequency distribution in the ***i***th niche.

The intensity of split in the population is 0 even if there is only one individual of beak size 1 that is chosen for mating. Runaway selection is also a possibility in this modeling setting, especially if the strength of selection is very high. Since there is no coexistence of two types of individuals when runaway selection occurs, the value of intensity of split is considered to be 0.

To study the effect of varying the strength of disruptive selection, niche widths (${\sigma }_{1}\& {\sigma }_{2}$) are altered. But, the width of one niche is always equal to the width of the other. Therefore, the width of each niche can be calculated easily using Eq. 1.

In this work, the choosiness of the females and the investment strategy of the males is capped at 0.4 and 0.8, respectively.

All results, unless specified otherwise, are based on the data obtained after simulations for 50 generations. An average of 50 repeats is reported, unless mentioned otherwise. All simulations were performed in MATLAB R2019a. The codes used in the study are provided in the Supplement.

### Reporting summary

Further information on research design is available in the Nature Research Reporting Summary linked to this article.

### Supplementary information

Supplement

Reporting summary

## Data Availability

The MATLAB codes used in this study are shared in the manuscript Supplement.
