# Supplementary material for: Ecological disruptive selection acting on quantitative loci can drive sympatric speciation
Source: NPJ Syst Biol Appl. 2024 Jan 15;10:6. doi: 10.1038/s41540-024-00332-w (PMC10789801; doi:10.1038/s41540-024-00332-w)
Supplement: Supplementary file 1 — Supplement [file 41540_2024_332_MOESM1_ESM.pdf]

Supplementary Material: Ecological disruptive selection acting on quantitative loci can drive sympatric speciation.

Pavithra Venkataraman[1,*] and Supreet Saini[1]

[1] Department of Chemical Engineering, Indian Institute of Technology Bombay, Mumbai, India 400 076

* Corresponding Author. Email: pavithrav@iitb.ac.in, Phone: +91 22 2576 4235

Running Title: When is sympatric speciation possible?

I. Effect of increase in disruptive selection (DS) and sexual selection (SS) on split intensity, beak size, choosiness and investment strategy.

A. Intensity of split

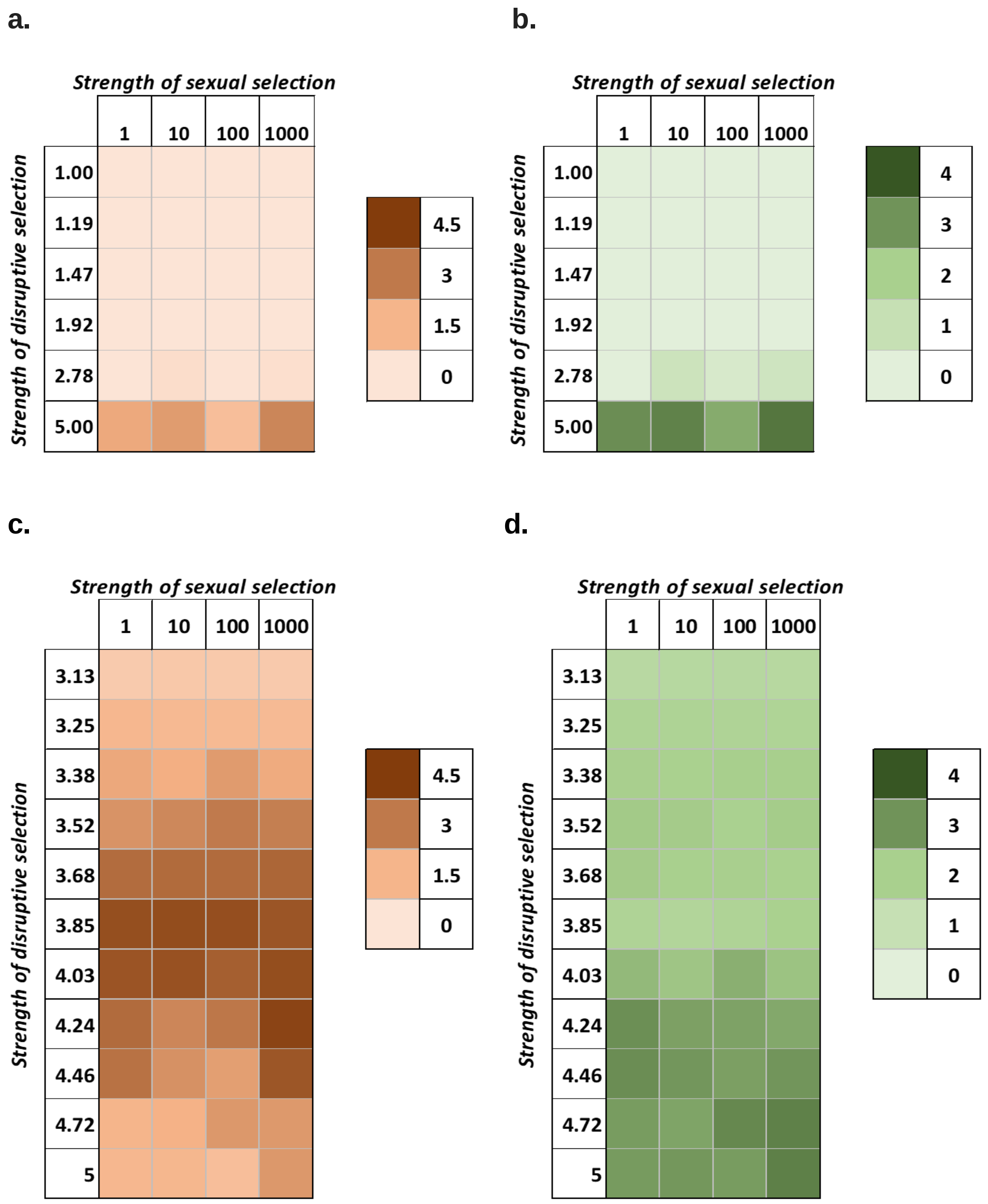

Supplementary Figure 1. These heat plots show the mean intensity of split (a and c, calculated as average obtained considering all the evolutionary outcomes) and its standard deviation (b and d) at the end of 50 generations, for 50 repeats. The number of loci controlling beak size, female choosiness, and male investment strategy were 20 each.

B. Beak size

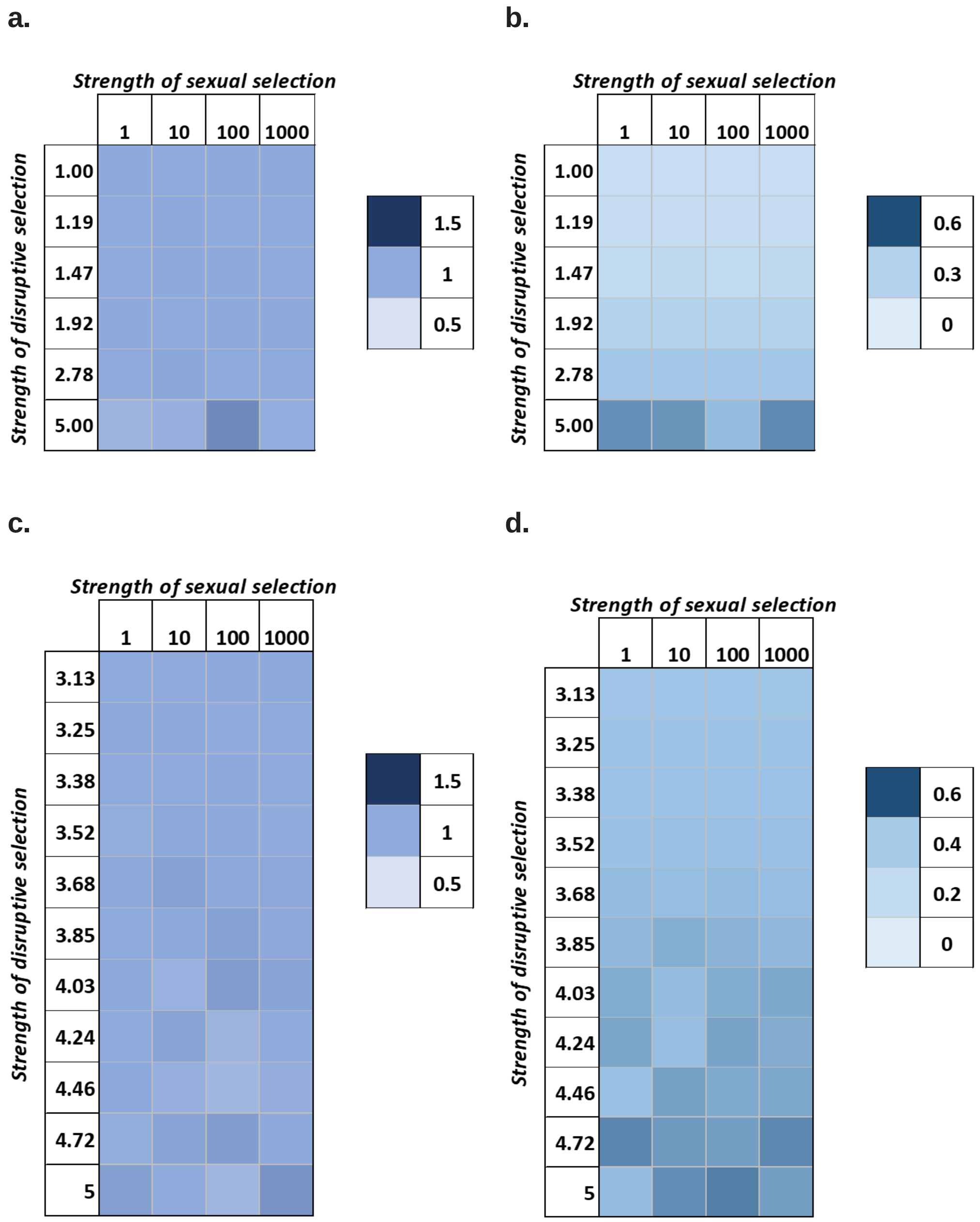

Supplementary Figure 2. These heat plots show the mean beak size (a and c, calculated as average obtained considering all the evolutionary outcomes) and its standard deviation (b and d) at the end of 50 generations, for 50 repeats. The number of loci controlling beak size, female choosiness, and male investment strategy were 20 each.

C. Choosiness

a.

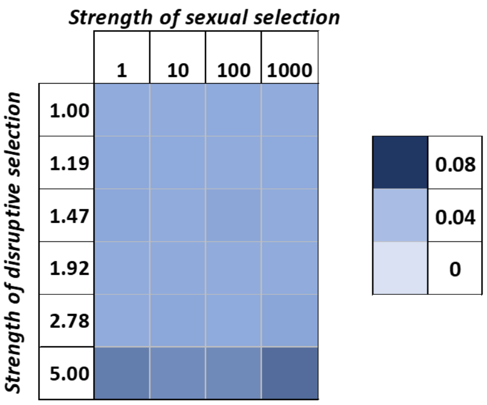

b.

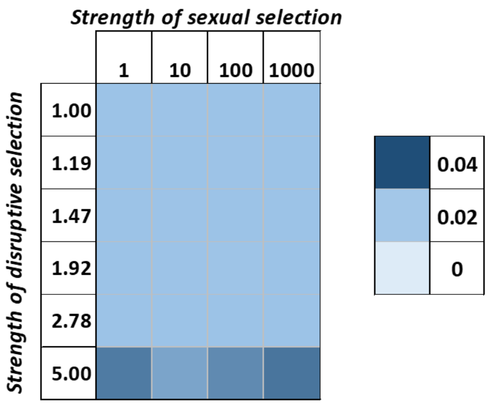

c.

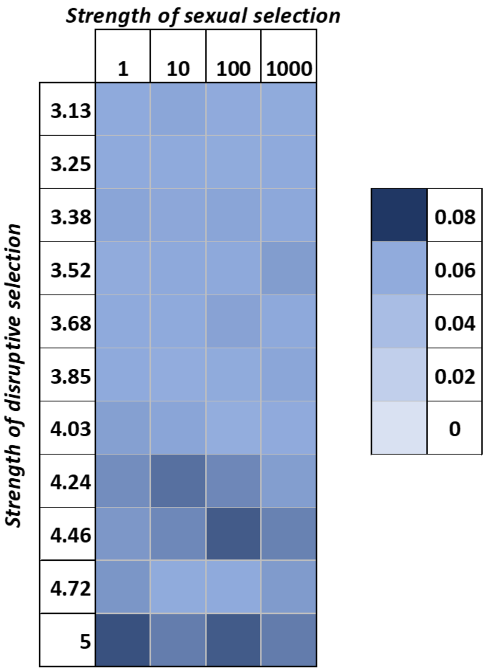

d.

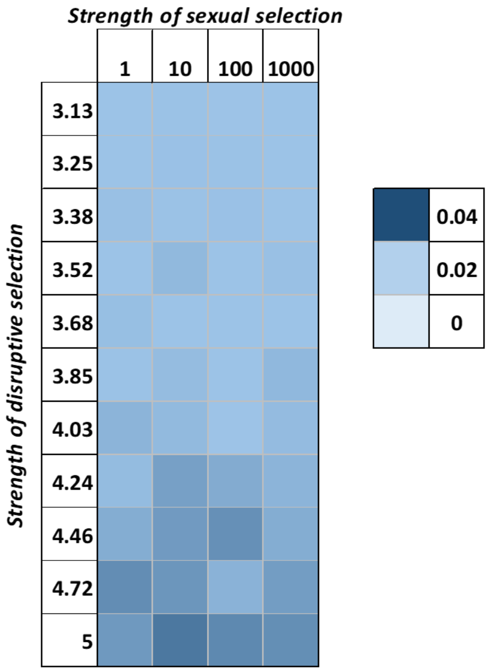

Supplementary Figure 3. These heat plots show the mean female choosiness (a and c, calculated as average obtained considering all the evolutionary outcomes) and its standard deviation (b and d) at the end of 50 generations, for 50 repeats. The number of loci controlling beak size, female choosiness, and male investment strategy were 20 each.

D. Investment strategy

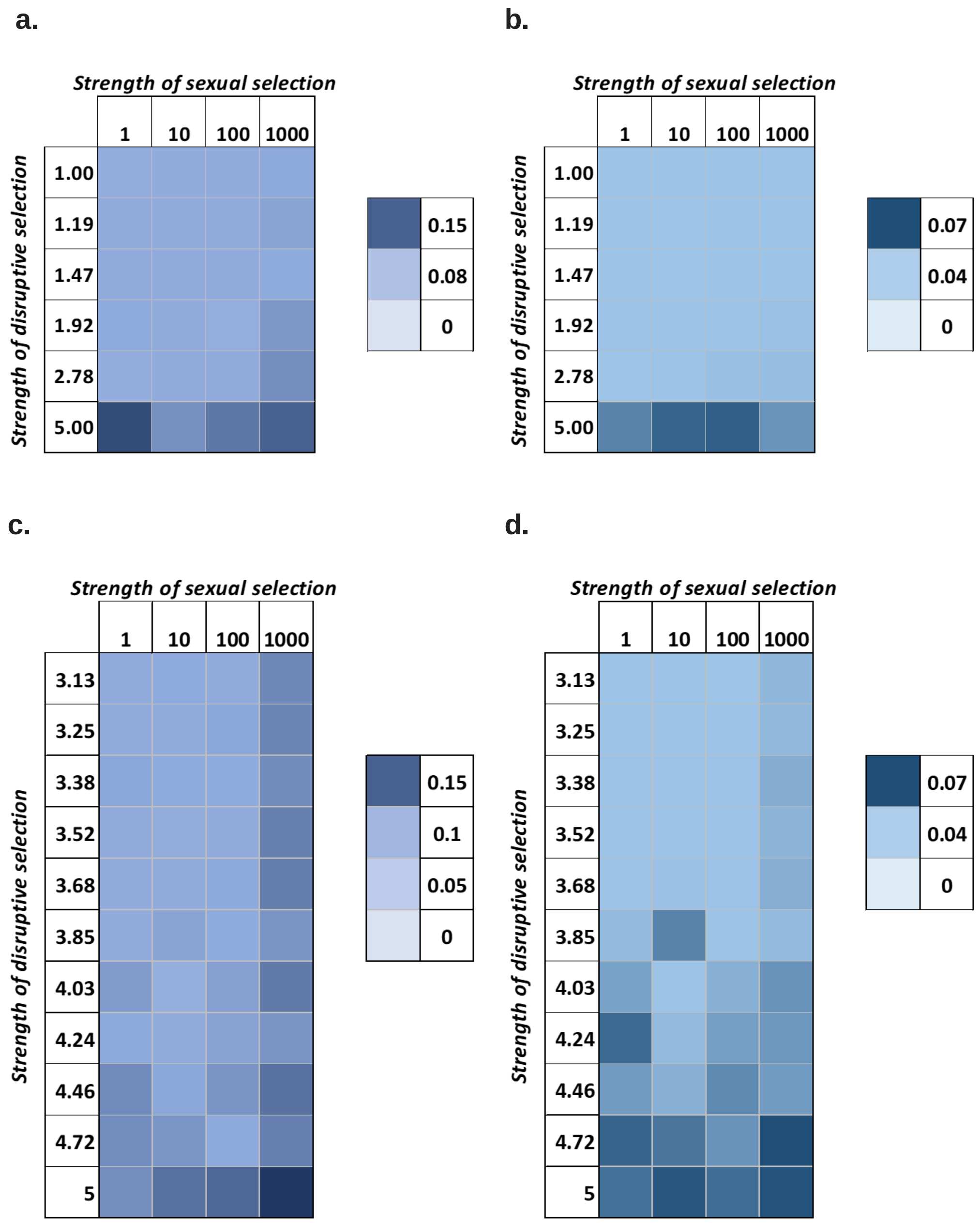

Supplementary Figure 4. These heat plots show the mean male investment strategy (a and c, calculated as average obtained considering all the evolutionary outcomes) and its standard deviation (b and d) at the end of 50 generations, for 50 repeats. The number of loci controlling beak size, female choosiness, and male investment strategy were 20 each.

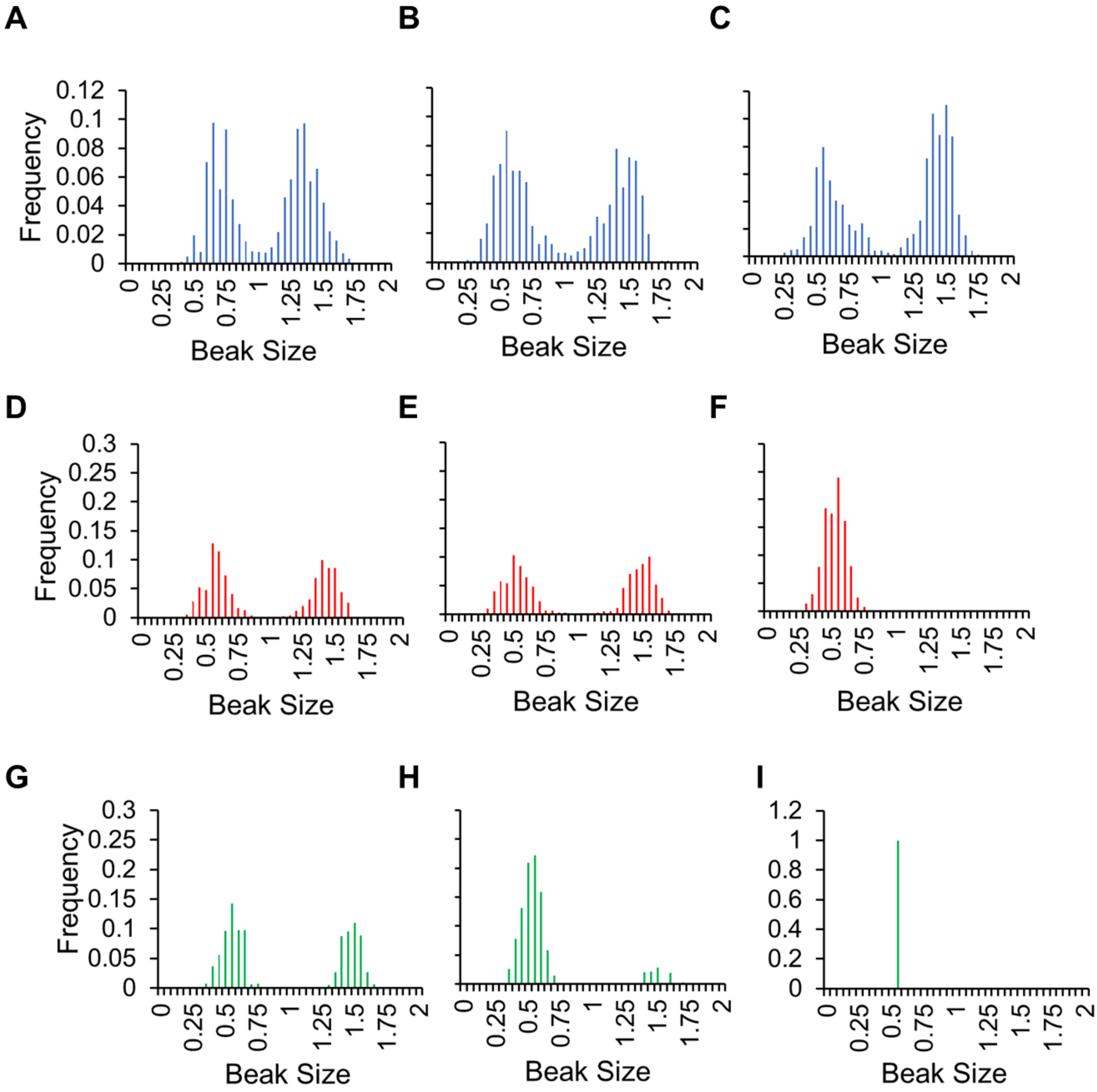

Supplementary Figure 5. Population dynamics for different strengths of disruptive selection. When the strength of ecological disruptive selection is low (2.94), the distribution of beak sizes of the individuals of the population is shown at 5 generations **(A)**, after 10 generations **(B)**, and after 50 generations **(C)**. When the strength of ecological disruptive selection is intermediate (3.85), the distribution of beak sizes of the individuals of the population is shown at 5 generations **(D)**, after 10 generations **(E)**, and after 50 generations **(F)**. When the strength of ecological disruptive selection is high (5.00), the distribution of beak sizes of the individuals of the population is shown at 5 generations **(G)**, after 10 generations **(H)**, and after 50 generations **(I)**.

No split condition: female choosiness and male investment strategy

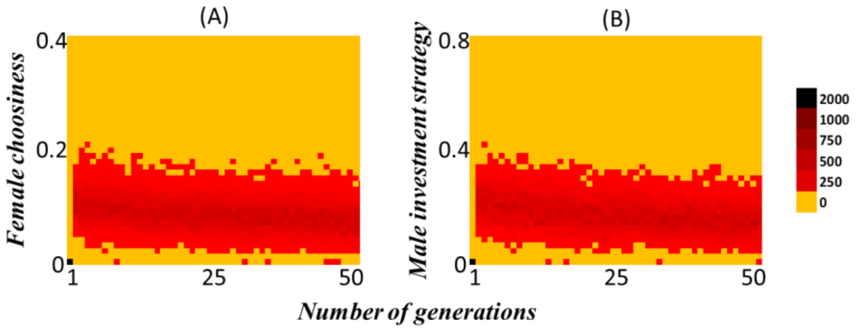

Runaway selection: Female choosiness and male investment strategy

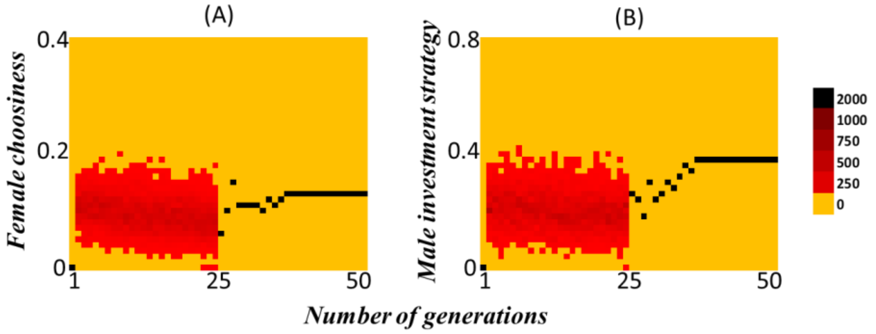

Supplementary Figure 6. Temporal variation in the traits under sexual selection. (Top) Variation in female choosiness **(A)** and male investment **(B)** in cases where the population does not split into two distinct groups at the end of 50 generations. **(Bottom)** Variation in female choosiness **(A)** and male investment **(B)** in cases where the population exhibits runaway selection.

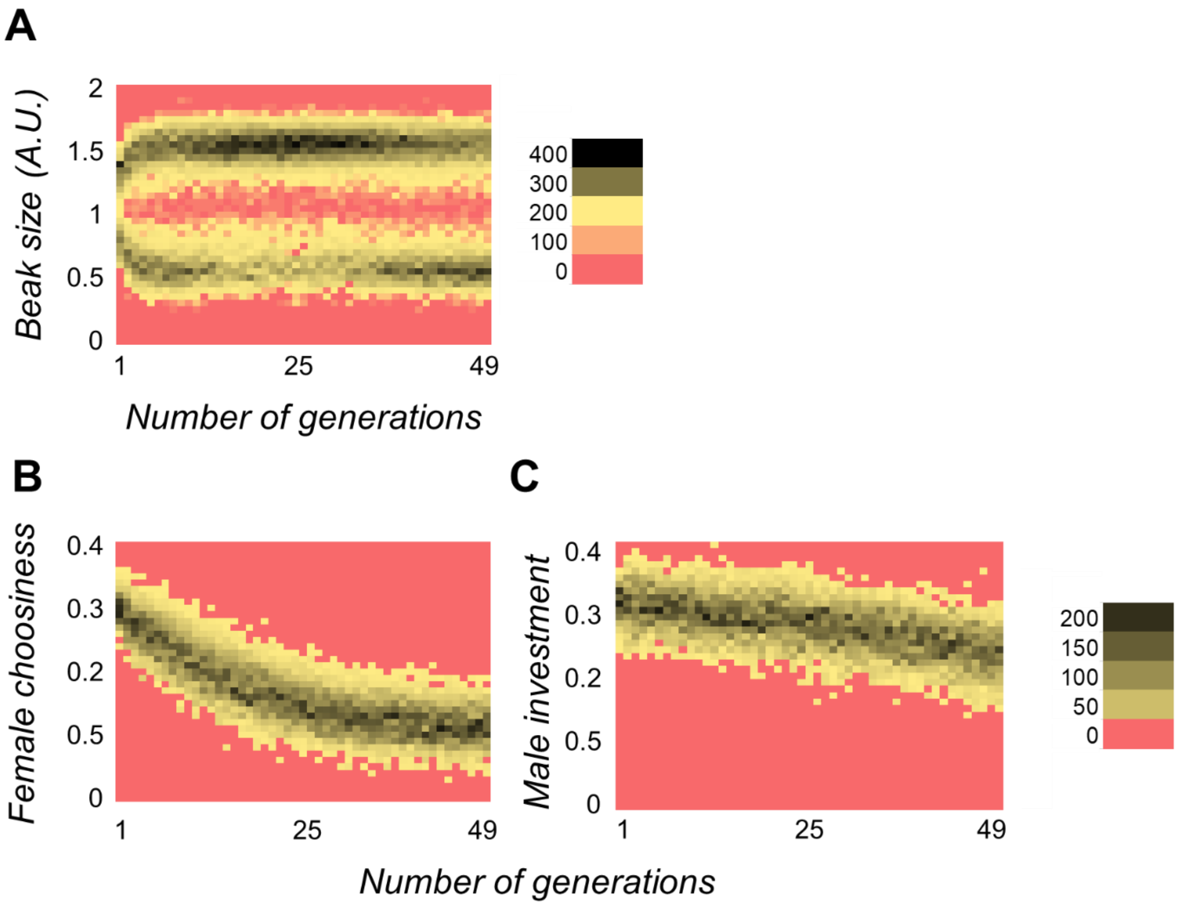

Supplementary Figure 7. Traits under sexual selection do not show divergence. In an environment in which the starting population's trait values of female choosiness and male investment strategy are maximum, we see that **(A)** the population splits into two distinct groups by the end of 50 generations. **(B)** and **(C)** show the temporal variation of female choosiness and male investment strategy, respectively. Given that a split in population occurs independent of these two trait values, they are said to be evolving neutrally.

II. Effect of altering genetic architecture on the intensity of split and beak size

<u>1. Null case</u>

A. Intensity of split

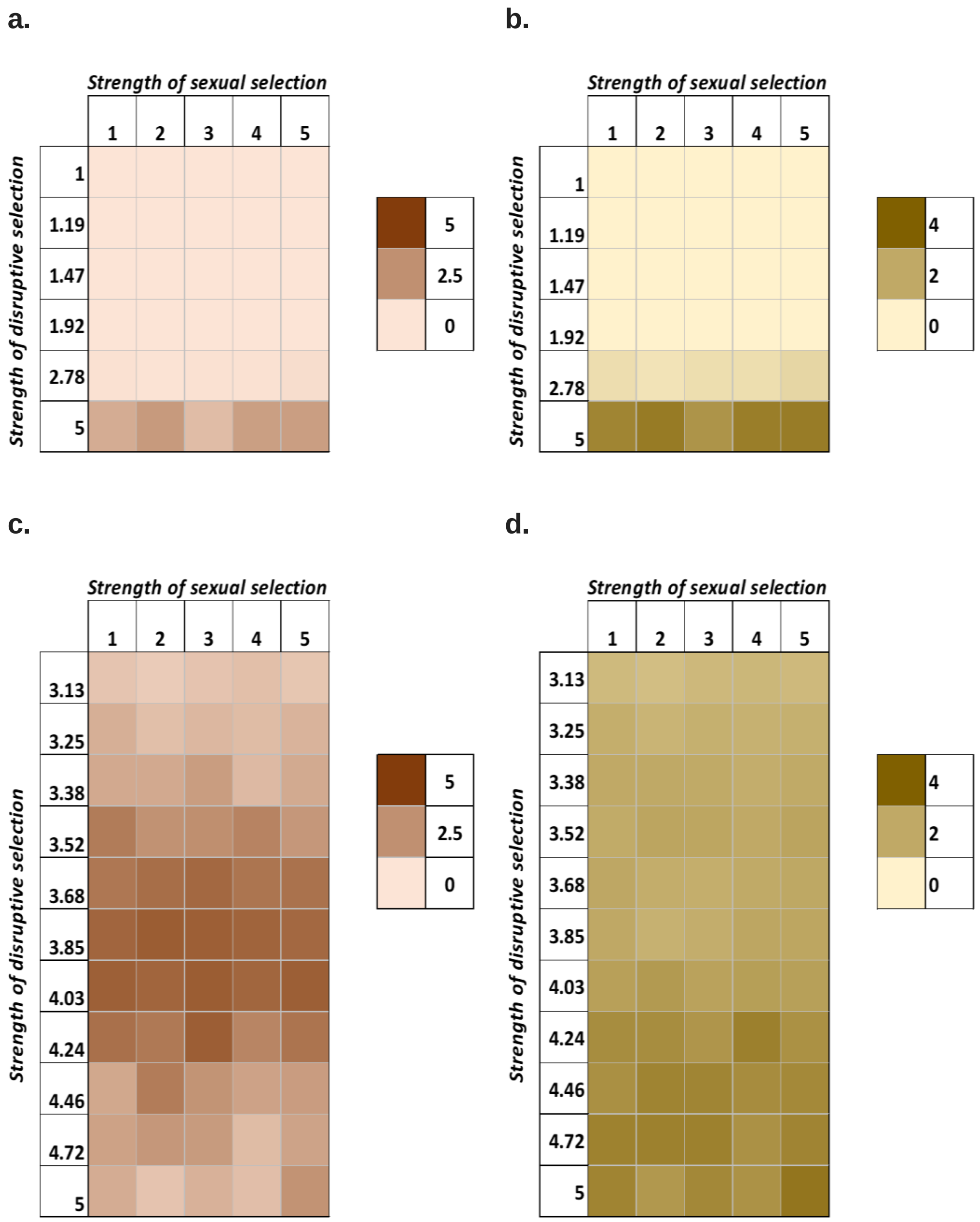

Supplementary Figure 8. These heat plots show the mean intensity of split (a and c, calculated as average obtained considering all the evolutionary outcomes) and its standard deviation (b and d) at the end of 50 generations, for 50 repeats. The number of loci controlling beak size, female choosiness, and male investment strategy were 20 each.

B. Beak size

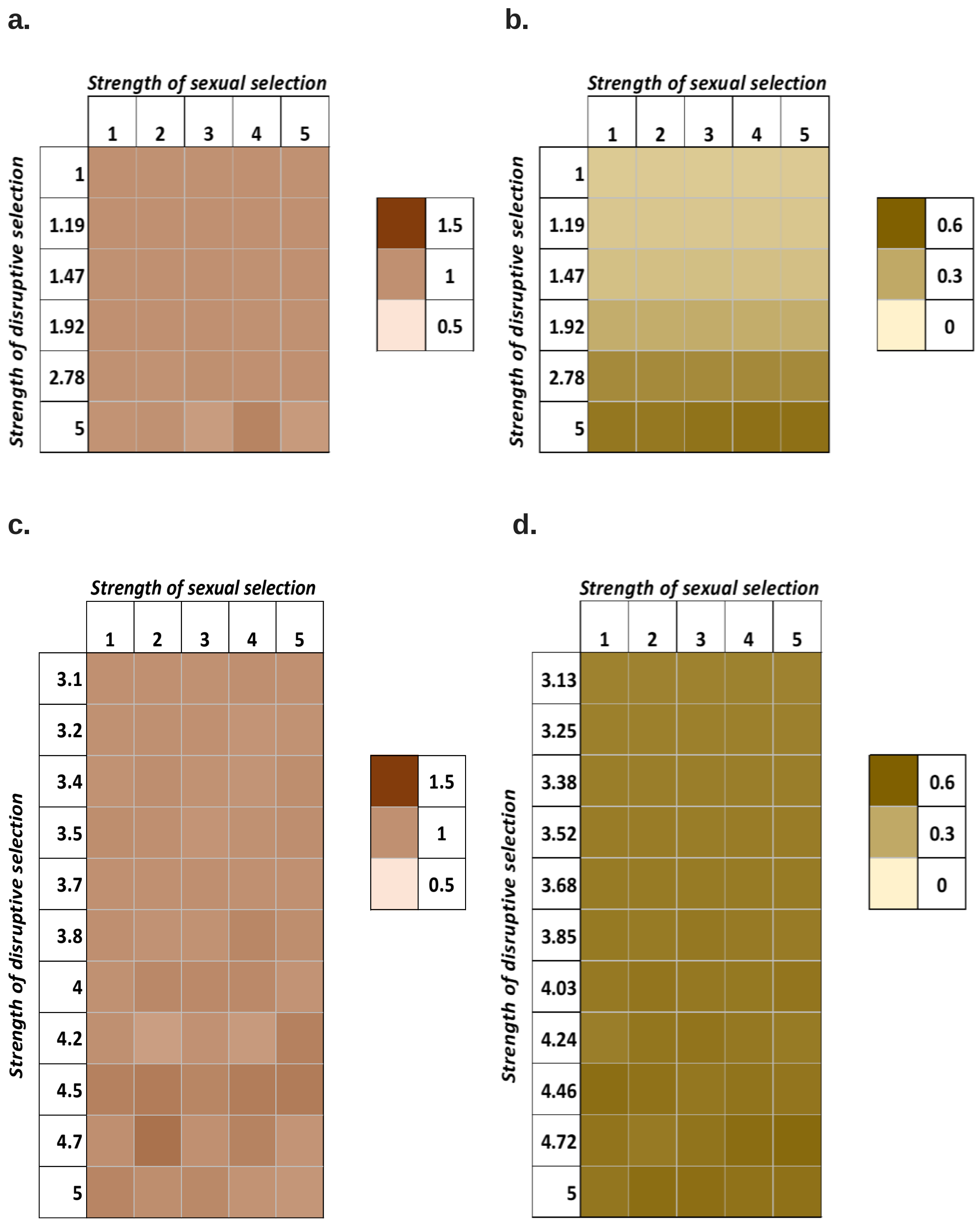

Supplementary Figure 9. These heat plots show the mean beak size (a and c, calculated as average obtained considering all the evolutionary outcomes) and its standard deviation (b and d) at the end of 50 generations, for 50 repeats. The number of loci controlling beak size, female choosiness, and male investment strategy were 20 each.

2. Dominance in the loci controlling beak size

A. Intensity of split

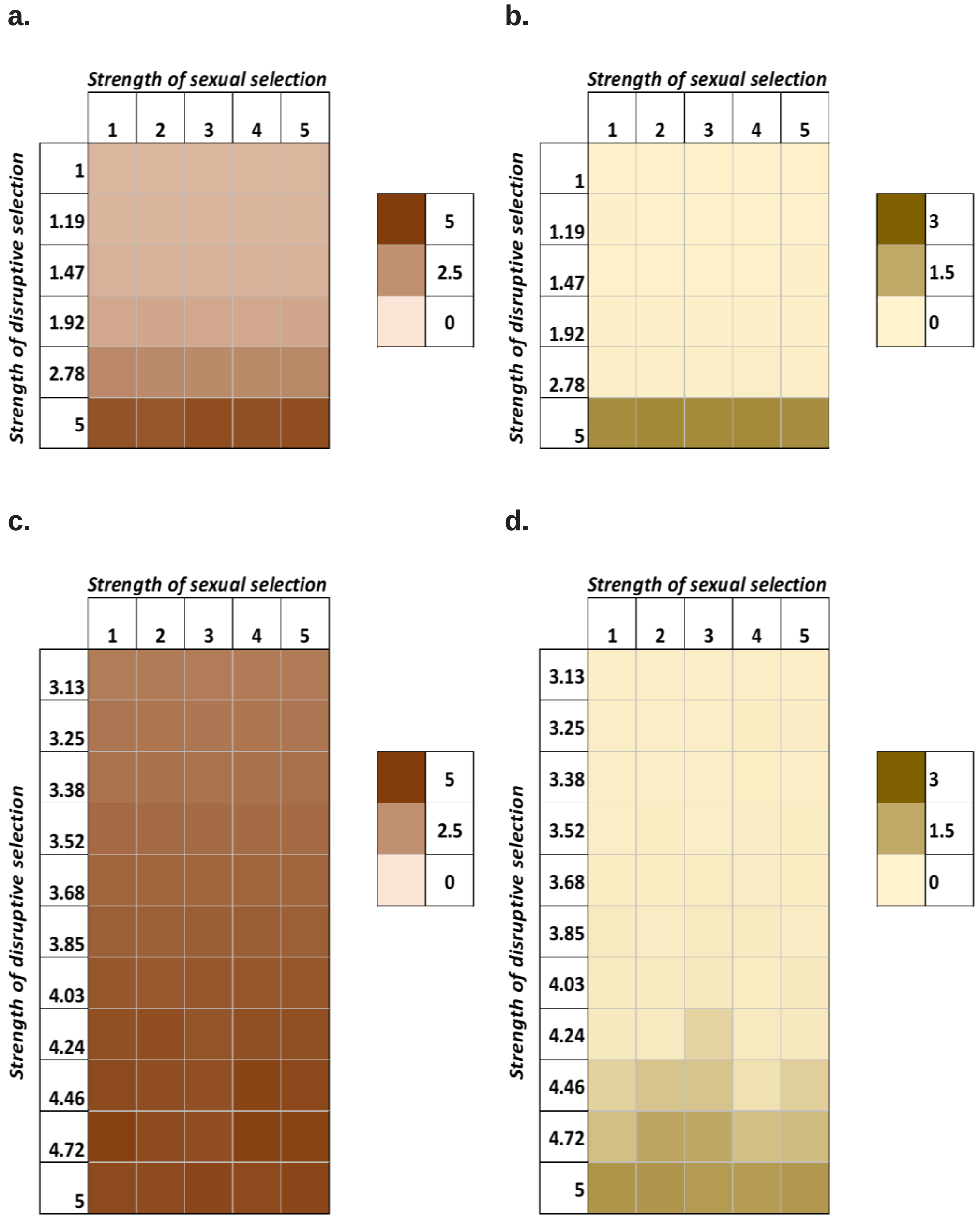

Supplementary Figure 10. These heat plots show the mean intensity of split (a and c, calculated as average obtained considering all the evolutionary outcomes) and its standard deviation (b and d) at the end of 50 generations, for 50 repeats. The number of loci controlling beak size, female choosiness, and male investment strategy were 20 each.

B. Beak size

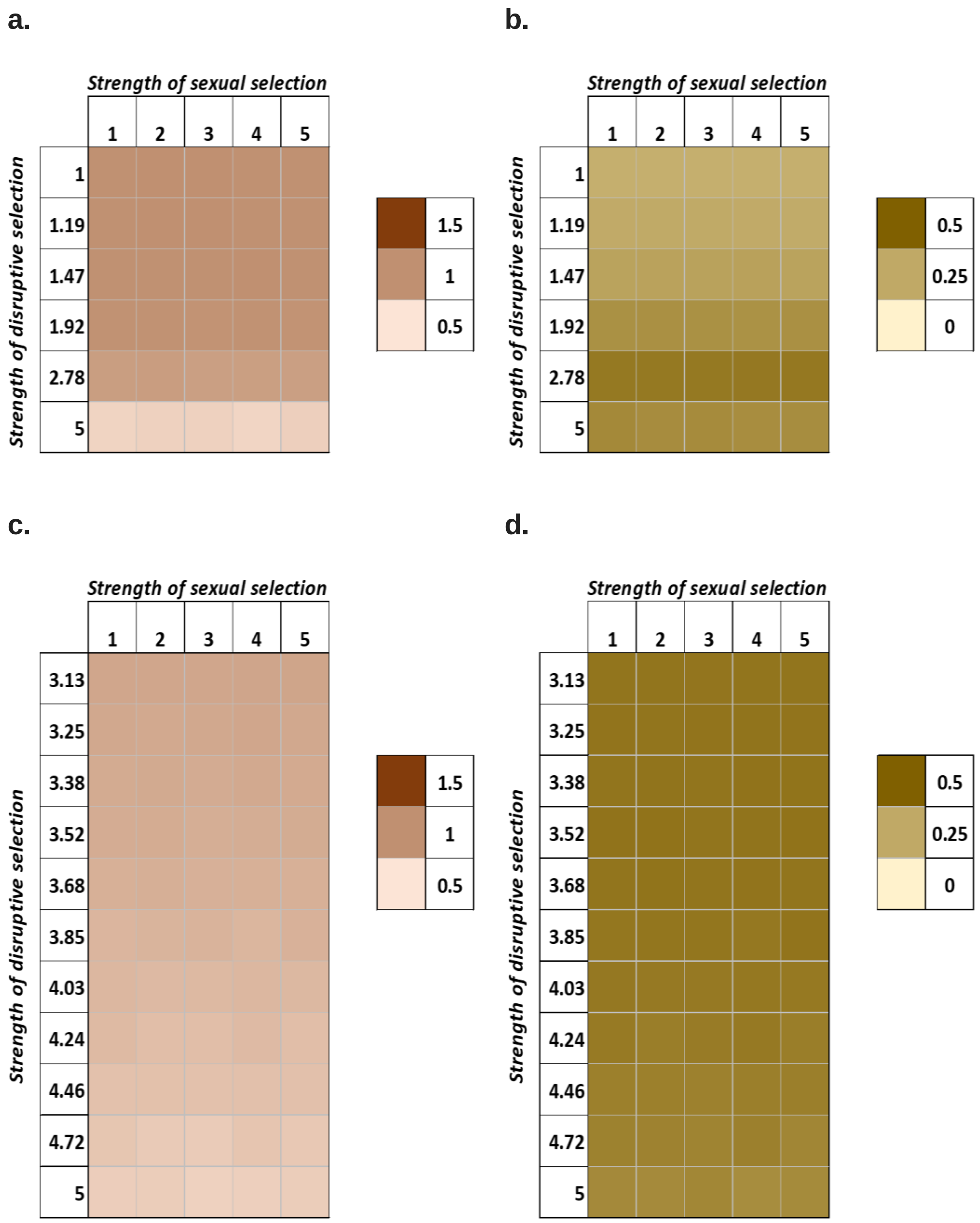

Supplementary Figure 11. These heat plots show the mean beak size (a and c, calculated as average obtained considering all the evolutionary outcomes) and its standard deviation (b and d) at the end of 50 generations, for 50 repeats. The number of loci controlling beak size, female choosiness, and male investment strategy were 20 each.

3. Unequal contribution of loci controlling beak size

A. Split

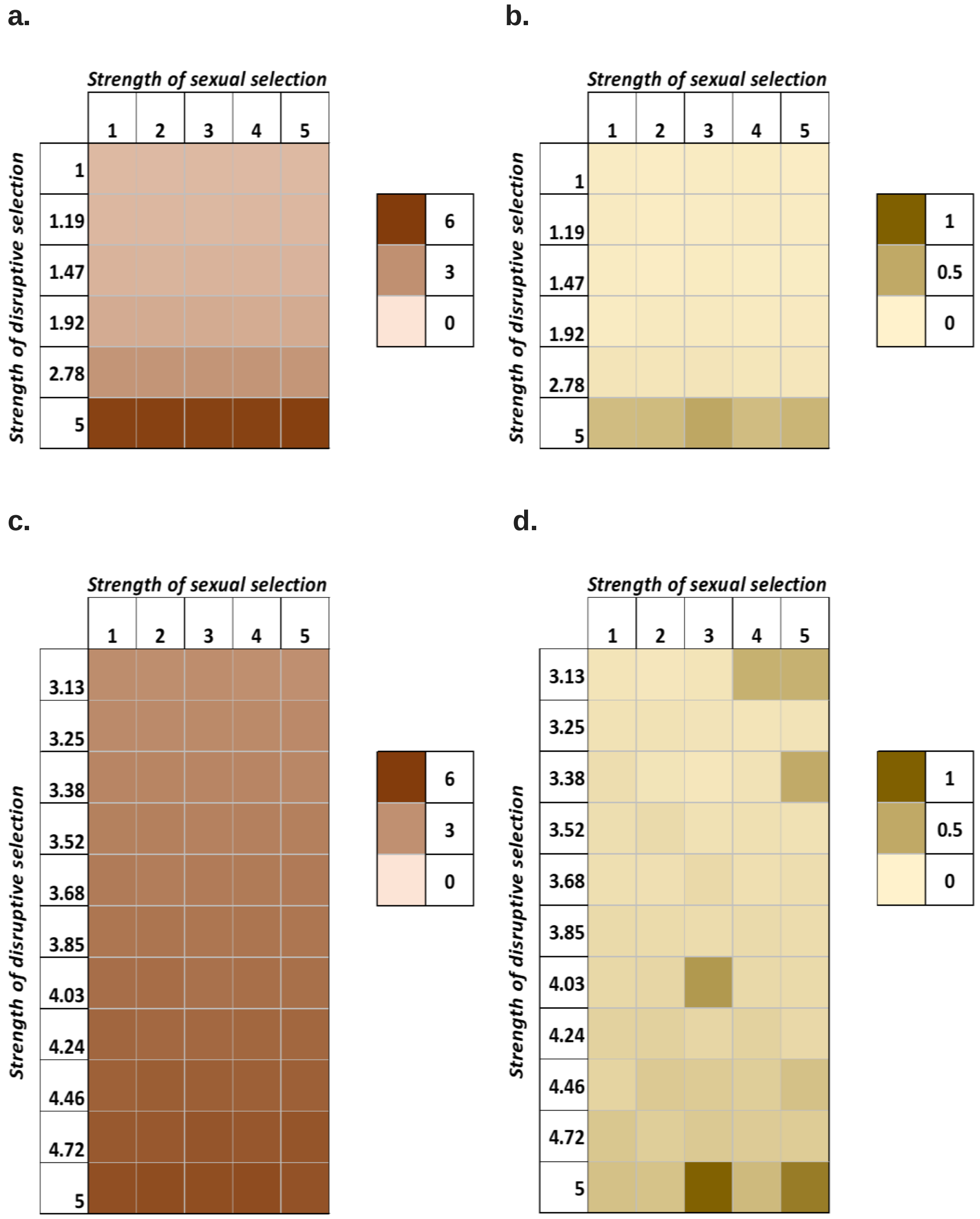

Supplementary Figure 12. These heat plots show the mean intensity of split (a and c, calculated as average obtained considering all the evolutionary outcomes) and its standard deviation (b and d) at the end of 50 generations, for 50 repeats. The number of loci controlling beak size, female choosiness, and male investment strategy were 20 each.

B. Beak size

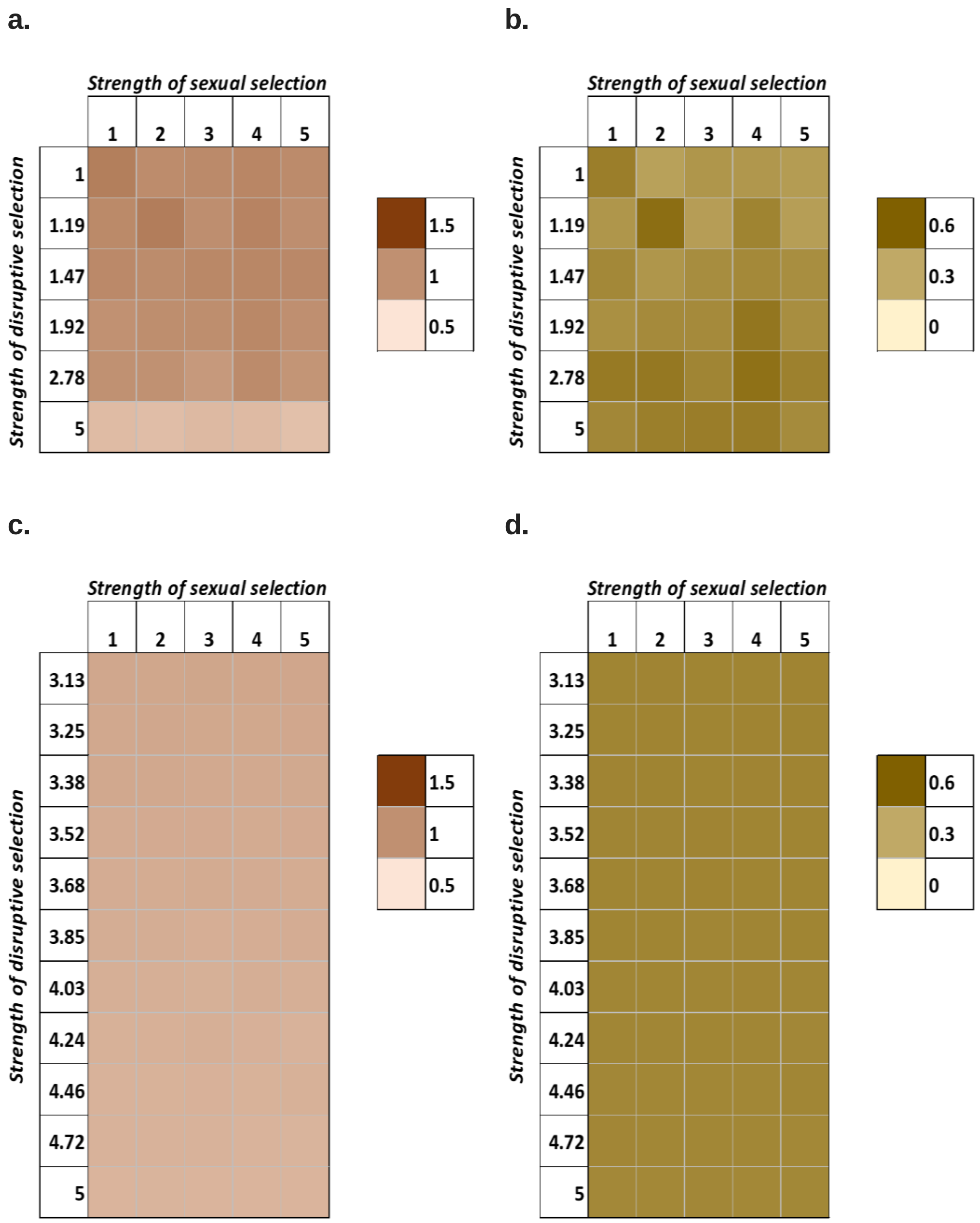

Supplementary Figure 13. These heat plots show the mean beak size (a and c, calculated as average obtained considering all the evolutionary outcomes) and its standard deviation (b and d) at the end of 50 generations, for 50 repeats. The number of loci controlling beak size, female choosiness, and male investment strategy were 20 each.

4. Unequally contribution loci of beak size that also show dominance relationships

A. Split

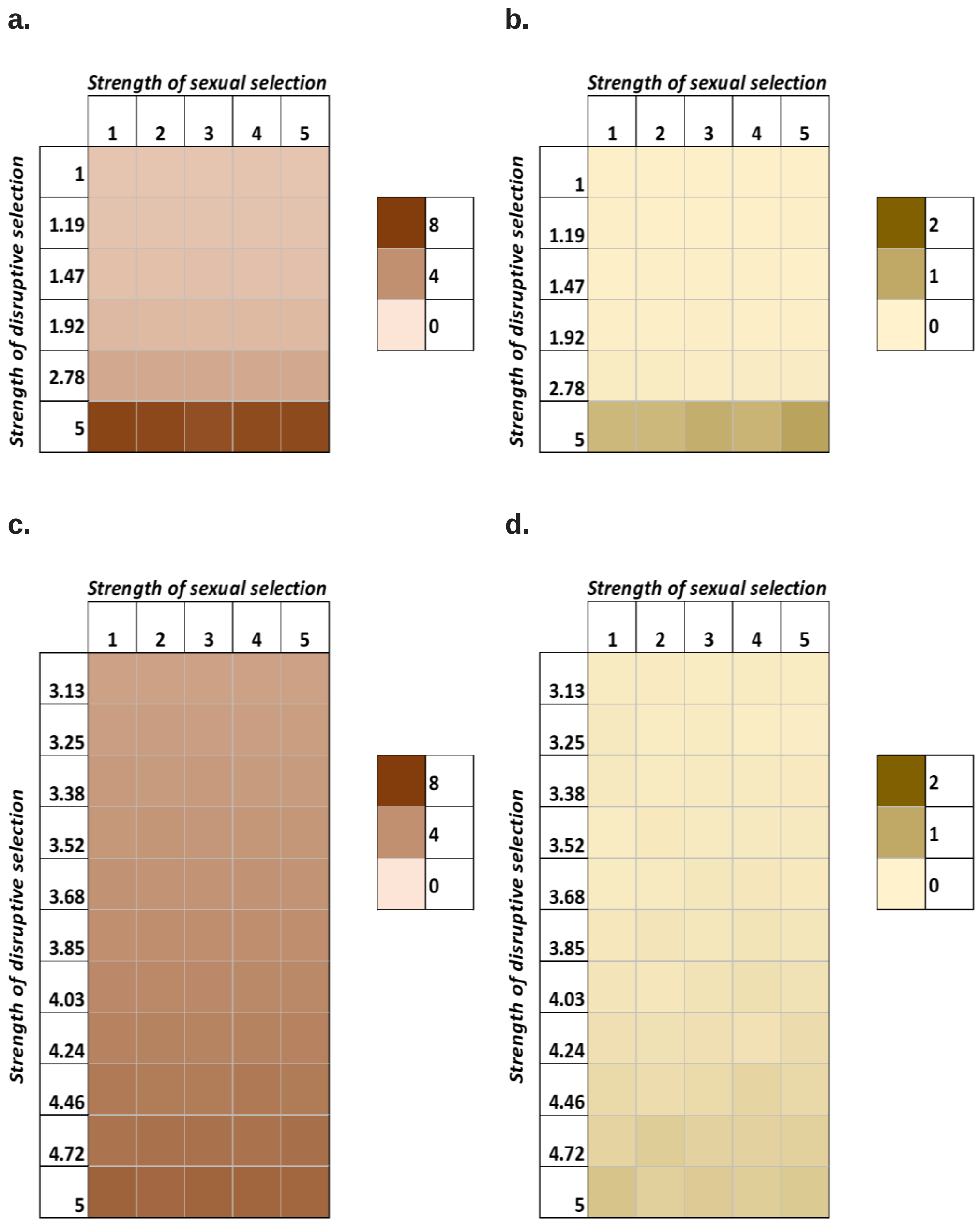

Supplementary Figure 14. These heat plots show the mean intensity of split (a and c, calculated as average obtained considering all the evolutionary outcomes) and its standard deviation (b and d) at the end of 50 generations, for 50 repeats. The number of loci controlling beak size, female choosiness, and male investment strategy were 20 each.

B. Beak size

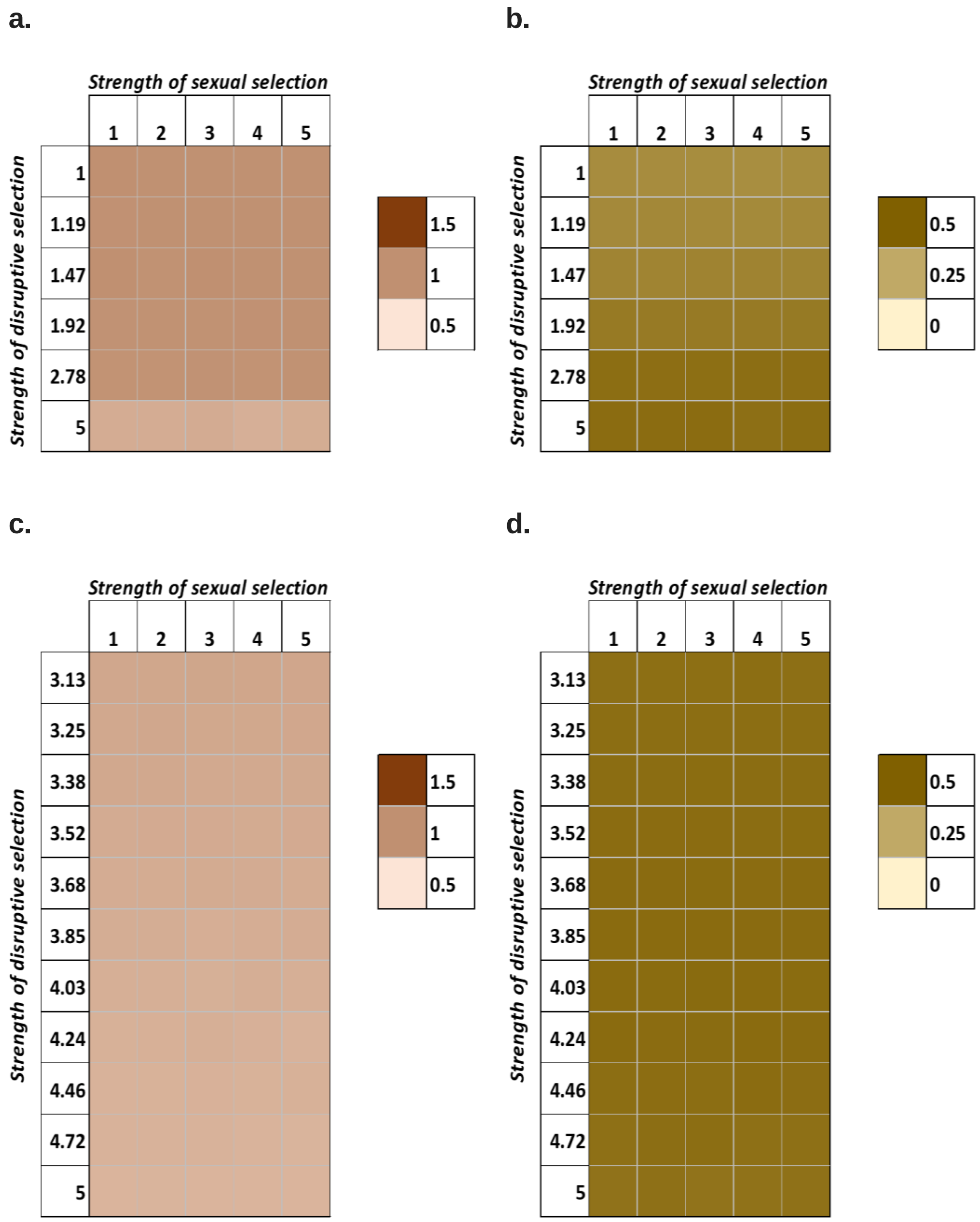

Supplementary Figure 15. These heat plots show the mean intensity of split (a and c, calculated as average obtained considering all the evolutionary outcomes) and its standard deviation (b and d) at the end of 50 generations, for 50 repeats. The number of loci controlling beak size, female choosiness, and male investment strategy were 20 each.

Codes

```
mainfile
clc
close all

dim1=1;
dim2=20; %number of loci controlling x,t, and p
xmu=2/(2*dim2); %contribution of each locus controlling x
tmu=0.8/(2*dim2); %contribution of each locus controlling t
pmu=0.4/(2*dim2); %contribution of each locus controlling p
totp=2*dim2*pmu; %maximum permissible choosiness

%unequal contributions of loci controlling x
uneqx1=exprnd(xmu,dim1,dim2);
uneqx=1*uneqx1/sum(uneqx1);

%dominance + unequal contribution of loci controlling x
uneqxdom11=exprnd(2*xmu,dim1,dim2/2);
uneqxdom1=uneqxdom11/sum(uneqxdom11);
uneqxdom12=exprnd(2*xmu,dim1,dim2/2);
uneqxdom2=uneqxdom12/sum(uneqxdom12);
uneqxdom=[uneqxdom1, uneqxdom2];

%unequal contribution of loci controlling p
uneqp1=exprnd(pmu,dim1,dim2);
uneqp=0.2*uneqp1/sum(uneqp1);

%dominance + unequal contribution of loci controlling x
uneqpdom11=exprnd(2*pmu,dim1,dim2/2);
uneqpdom1=uneqpdom11/sum(uneqpdom11);
uneqpdom12=exprnd(2*pmu,dim1,dim2/2);
uneqpdom2=uneqpdom12/sum(uneqpdom12);
uneqpdom=0.2*[uneqpdom1, uneqpdom2];

%unequal contribution of loci controlling t
uneqt1=exprnd(tmu,dim1,dim2);
uneqt=0.4*uneqt1/sum(uneqt1);

%dominance + unequal contribution of loci controlling t
uneqtdom11=exprnd(2*tmu,dim1,dim2/2);
uneqtdom1=uneqtdom11/sum(uneqtdom11);
uneqtdom12=exprnd(2*tmu,dim1,dim2/2);
uneqtdom2=uneqtdom12/sum(uneqtdom12);
uneqtdom=0.4*[uneqtdom1, uneqtdom2];

%computation of values of intensity of split
spec=zeros(1,1,16);
dev_split=zeros(1,1,16);
beaks=zeros(1,1,16);
choose=zeros(1,1,16);
invest=zeros(1,1,16);
dev_beaks=zeros(1,1,16);
dev_choose=zeros(1,1,16);
dev_invest=zeros(1,1,16);
```

```
page=1;
for i=1:4
    for j=1:4
        for k=1:4
            if i==1
               bi=2;
               dellx=xmu;
       else if i==2
            bi=1;
            dellx=2*xmu;
       else if i==3
                bi=2;
                dellx=uneqx;
       else if i==4
                 bi=1;
                 dellx=uneqxdom;
                end
            end
        end
            end
    if j==1
        pi=2;
        dellp=pmu;
        sump=0.4;
    else if j==2
            pi=1;
            dellp=2*pmu;
            sump=0.4;
        else if j==3
                pi=2;
                dellp=uneqp;
                sump=sum(uneqp);
            else if j==4
                    pi=1;
                    dellp=uneqpdom;
                    sump=sum(uneqpdom);
                end
            end
        end
    end
         if k==1
        ti=2;
        dellt=tmu;
    else if k==2
            ti=1;
            dellt=2*tmu;
        else if k==3
                ti=2;
                dellt=uneqt;
            else if k==4
                    ti=1;
                    dellt=uneqtdom;
                end
            end
           end
```

```
            end

[spec1,splitstdev,bavg,pavg,tavg,devb,devp,devt]=qfic(dim1,dim2,dell
x,dellp,dellt,sump,bi,pi,ti);
        spec(:,:,page)=spec1;
        dev_split(:,:,page)=splitstdev;
        beaks(:,:,page)=bavg;
        choose(:,:,page)=pavg;
        invest(:,:,page)=tavg;
        dev_beaks(:,:,page)=devb;
        dev_choose(:,:,page)=devp;
        dev_invest(:,:,page)=devt;
        page=page+1;
        end

    end
end


function[split,stdevsplit,avgbeak,avgp,avgt,stdev_beak,stdev_p,stdev
_t]=qfic(sz1,sz2,xdell,pdell,tdell,psumm,ib,ip,it)

%width of distribution of resources
stdev1=0.13;
stdev2=0.13;

n1=length(stdev1);

%strength of sexual selection
stsex=5;
n2=length(stsex);

split=zeros(n1,n2);
sep=zeros(n1,n2);
itersep=zeros(n1,n2,50);
beaks=zeros(2000,50);
choosiness=zeros(1000,50);
investments=zeros(1000,50);

divsel=zeros(n1,1);

for i=1:n1
    devst1=stdev1(i);
    devst2=stdev2(i);
    divsel(i)=1/(devst1+devst2); %strength of disruptive selection
     for j=1:n2
        alp=stsex(j); %strength of sexual selection
        iter=1;
        while iter<=50
        [sep(i,j),finalbeaks,pees,tees]=
symsp(devst1,devst2,alp,xdell,pdell,tdell,psumm,sz2,ib,ip,it);
        spval(iter)=sep(i,j);
        devt(iter)=std(tees);
        meant(iter)=mean(tees);

        for m=1:n1
```

```
            for n=1:n2
                if sep(m,n)<0
                    sep(m,n)=0;
                end
            end
        end

        split(i,j)=split(i,j)+sep(i,j);
        itersep(i,j,iter)=sep(i,j);
        beaks(:,iter)=finalbeaks;
        choosiness(:,iter)=pees;
        investments(:,iter)=tees;
        iter=iter+1;
        end

avgbeak(i,j)=mean(mean(beaks)');
bavg=avgbeak(i,j);
avgp(i,j)=mean(mean(choosiness)');
pavg=avgp(i,j);
avgt(i,j)=mean(mean(investments)');
tavg=avgt(i,j);

sumb=0;
sumt=0;
sump=0;

for m=1:2000
    for n=1:50
        sumb=sumb+((beaks(m,n)-bavg)^2);
    end
end

for m=1:1000
    for n=1:50
        sumt=sumt+((investments(m,n)-tavg)^2);
        sump=sump+((choosiness(m,n)-pavg)^2);
    end
end

stdev_beak(i,j)=sqrt(sumb/(2000*50));
stdev_t(i,j)=sqrt(sumt/(1000*50));
stdev_p(i,j)=sqrt(sump/(1000*50));
     end
end

stdevsplit=std(itersep,0,3);
split=split/50;
end

function[diff,matxf,choosi,invest]=symsp(sig1,sig2,alpha,delx,delp,d
elt,sump,nloci,bi,pi,ti)

nind=1000;  %number of individuals
ngenerations=50; %number of generations

matm=zeros(nind,1); %array of males that mate%
```

```
matf=zeros(nind,1); %array of females that mate%

xtrack=zeros(ngenerations,nind); %beak size of individuals of every
generation%
ttrack=zeros(ngenerations,nind/2); %investment strategies of the
males of every generation%
ptrack=zeros(ngenerations,nind/2); %choosiness of the females of
every generation%
mtrack=zeros(ngenerations,nind); %investment strategy of males of
every generation
ftrack=zeros(ngenerations,nind); %choosiness of females of every
generation

fpcr1=zeros(nind/2,nloci); %first strand of female's gene - p
fpcr2=zeros(nind/2,nloci); %second strand of female's gene - p
mpcr1=zeros(nind/2,nloci); %first strand of male's gene - p
mpcr2=ones(nind/2,nloci); %second strand of male's gene - p

ftcr1=zeros(nind/2,nloci); %first strand of female's gene - t
ftcr2=ones(nind/2,nloci); %second strand of female's gene - t
mtcr1=zeros(nind/2,nloci); %first strand of male's gene - t
mtcr2=zeros(nind/2,nloci); %second strand of male's gene - t

if bi==2
fxcr1=zeros(nind/2,nloci); %first strand of female's gene - x
fxcr2=ones(nind/2,nloci); %second strand of female's gene - x

mxcr1=zeros(nind/2,nloci); %first strand of male's gene - x
mxcr2=ones(nind/2,nloci); %second strand of male's gene - x


else if bi==1
fxcr1=zeros(nind/2,nloci); %first strand of female's gene - x
fxcr2=zeros(nind/2,nloci); %second strand of female's gene - x
mxcr1=zeros(nind/2,nloci); %first strand of male's gene - x
mxcr2=zeros(nind/2,nloci); %second strand of male's gene - x
%altered genetic makeup in case of dominance
fxcr2(1:nind/2,1:nloci/2)=1;
mxcr2(1:nind/2,((nloci/2)+1):nloci)=1;
end
end

%means of normal distributions centered that indicate disruptive
selection%
mean1=0.5;
mean2=1.5;

for i=1:ngenerations

    ngen(i)=i;


[ff,fm,xavg,xvar,xtot,np1,np2,fx,mx]=fcalc(fxcr1,fxcr2,nind,mxcr1,mx
cr2,nloci,delx,mean1,mean2,sig1,sig2,bi); %function that calculates
beak size and fitness of the individuals
    avgxx(i)=xavg;
```

```
    varxx(i)=xvar;
    fitness=cat(1,ff,fm);
    avgf(i)=mean(fitness);

    femfit=ff;
    malfit=fm;

    [fp]=pcalc(fpcr1,fpcr2,nind,nloci,delp,pi); %function that
calculates choosiness of every female%
    avgp(i)=mean(fp);

    %calculation of probability that a female finds a partner based
on its
    %fitness and choosiness
    fcost=(exp(-8*fp.*fp.*fp/(sump)^3)).*femfit';
    sumfcost=sum(fcost);
    fprob1=fcost/sumfcost;

    sumf=0;
    for x=1:nind/2
        sumf=sumf+fprob1(x);
        fprob(x)=sumf;
    end

    [mt]=tcalc(mtcr1,mtcr2,nind,nloci,delt,ti); %function that
calculates investment strategy of every male%
    avgt(i)=mean(mt);

    % calculation of probability that a male escapes predation%
    for jj=1:nind/2
        predprop(jj)=exp(-mt(jj)/(1-mt(jj)));
    end

    summcost=sum(predprop);
    predprob=predprop/summcost;
    summ=0;
    for y=1:nind/2
        summ=summ+predprob(y);
        mprob(y)=summ;
    end

    %assignment of beak sizes, males' investment strategy and
females'
    %choosiness
    if i>=1
        xtrack(i,:)=xtot;
        ttrack(i,:)=mt;
        ptrack(i,:)=fp;
    end

    %meeting and mating event determination
    for p=1:nind
        r1=rand;
        ii=1;
        while r1>fprob(ii)
            ii=ii+1;
```

```
        end
        matf(p)=ii;

        if i==ngenerations
        choosi(p)=fp(ii);
        end

        for q=1:nind/2
            fdep(q)=(1-(mt(q)))*malfit(q)*mprob(q); %product of the
selected female's fitness with all the males' fitness
            matingp(q)=exp(mt(q)*malfit(q)*fp(ii)*alpha); %mating
probability of the selected female with all the males in the
population
        end
            sumfdep=sum(fdep);
            fdepp=fdep/sumfdep;
            summatp=sum(matingp);
            probmating=matingp/summatp;

            sumffdep=0;
            summating=0;

            for pp=1:nind/2
              sumffdep=sumffdep+fdepp(pp);
              fitnessprob(pp)=sumffdep;
              summating=summating+probmating(pp);
              matprb(pp)=summating;
            end

        iiii=1;
        while iiii==1
            r2=rand;
            r3=rand;
            iii=1;
         while r2>fitnessprob(iii)
            iii=iii+1;
         end
         ev=iii;
         if r3>matprb(ev)
            iiii=1;
         else
             iiii=0;
         end
        end
        matm(p)=ev;
        if i==ngenerations
        invest(p)=mt(ev);
        end
    end

    for b=1:nind
        indf=matf(b);
        indm=matm(b);
        matfemx(b)=fx(indf);
        matmalx(b)=mx(indm);
    end
```

```
    if i>=1
        mtrack(i,:)=matmalx;
        ftrack(i,:)=matfemx;
    end



    %determination of the genotypes of the offspring produced by the
mating event%

[fempcr1,fempcr2,malpcr1,malpcr2]=pmat(fpcr1,fpcr2,mpcr1,mpcr2,nind,
nloci,matf,matm);

[femtcr1,femtcr2,maltcr1,maltcr2]=tmat(ftcr1,ftcr2,mtcr1,mtcr2,nind,
nloci,matf,matm);

[femxcr1,femxcr2,malxcr1,malxcr2]=xmat(fxcr1,fxcr2,mxcr1,mxcr2,nind,
nloci,matf,matm);

    %assignment of female and male genotypes of the next generation%
    fpcr1=fempcr1;
    fpcr2=fempcr2;
    mpcr1=malpcr1;
    mpcr2=malpcr2;

    ftcr1=femtcr1;
    ftcr2=femtcr2;
    mtcr1=maltcr1;
    mtcr2=maltcr2;

    fxcr1=femxcr1;
    fxcr2=femxcr2;
    mxcr1=malxcr1;
    mxcr2=malxcr2;
end

femxf=ftrack(ngenerations-1,:);
malxf=mtrack(ngenerations-1,:);
matxf=[femxf malxf];
femalep=ptrack(50,:);
malet=ttrack(50,:);

%calculation of intensity of split
diff=1;
bs1=0;
for pp=1:(2*nind)
    if matxf(pp)==1
        bs1=bs1+1;
    else
        bs1=bs1+0;
    end
end

if bs1~=0
    diff=0;
```

```
else
nsmallx=0;
nbigx=0;
 for hh=1:(nind*2)
     if matxf(hh)<1
         nsmallx=nsmallx+1;
     else if matxf(hh)>1
             nbigx=nbigx+1;
     end
     end
 end

  if nsmallx==0 && nbigx~=0
    diff=-1;
  else if nsmallx~=0 && nbigx==0
          diff=-1;
  else if nsmallx<2 || nbigx<2
          diff=-1;
  else if (nsmallx~=0 && nbigx~=0) && (nsmallx>2 && nbigx>2)

     smallx=zeros(nsmallx,1);
     bigx=zeros(nbigx,1);

     fmatx=sort(matxf);

     smallx=(fmatx(:,1:nsmallx))';
     bigx=(fmatx(:,(nsmallx+1):end))';

     pd1=fitdist(smallx,'Normal');
     m1=mean(pd1);
     dev1=std(pd1);
     pd2=fitdist(bigx,'Normal');
     m2=mean(pd2);
     dev2=std(pd2);

     diff=(m2-m1)/(dev2+dev1);
  end
  end
  end
  end
end

end

function[fitf,fitm,avgx,varx,totx,p1,p2,xf,xm]=fcalc(xfcr1,xfcr2,ind
n,xmcr1,xmcr2,loci,xdel,meano,meant,sigma1,sigma2,ib)

xfcr=zeros(indn/2,loci);
xmcr=zeros(indn/2,loci);

%calculation of beak size of the females:

for ll=1:indn/2
for l=1:loci
    if xfcr1(ll,l)==1 && xfcr2(ll,l)==1
```

```
        xfcr(ll,l)=ib;
    else if xfcr1(ll,l)==1 && xfcr2(ll,l)==0
            xfcr(ll,l)=1;
    else if xfcr1(ll,l)==0 && xfcr2(ll,l)==1
            xfcr(ll,l)=1;
    else if xfcr1(ll,l)==0 && xfcr2(ll,l)==0
            xfcr(ll,l)=0;
    end
    end
    end
    end
end
end
xf1=xdel.*xfcr;
xf=sum(xf1');

%calculation of beak size of the males:

for mm=1:indn/2
for m=1:loci
    if xmcr1(mm,m)==1 && xmcr2(mm,m)==1
        xmcr(mm,m)=ib;
    else if xmcr1(mm,m)==1 && xmcr2(mm,m)==0
            xmcr(mm,m)=1;
    else if xmcr1(mm,m)==0 && xmcr2(mm,m)==1
            xmcr(mm,m)=1;
    else if xmcr1(mm,m)==0 && xmcr2(mm,m)==0
            xmcr(mm,m)=0;
    end
    end
    end
    end
end
end
xm1=xdel.*xmcr;
xm=sum(xm1');

totx=[xf xm];

avgx=mean(totx);
varx=var(totx);

%calculation of number of individuals in a niche

p1=0;
p2=0;
for k=1:indn
    diff(k)=totx(k)-1;
    dif(k)=round(diff(k),6);
    if dif(k)<0
        p1=p1+1;
    else if dif(k)>0
            p2=p2+1;
    else if dif(k)==0
            p1=p1+0;
            p2=p2+0;
```

```
    end
    end
    end
end
nn1=p1;
nn2=p2;

intp=((sqrt(2)*meant)+(sqrt(2)*meano))/(sqrt(2)+sqrt(2));
fitintp=exp(-(intp-meano)^2/(2*sigma1*sigma1));

ff=zeros(indn/2,1);
fm=zeros(indn/2,1);

%calculation of fitness of the females
for i=1:indn/2
    xfeff(i)=round(xf(i),6);

    if xfeff(i)<1
        ff(i)=exp(-((xf(i)-meano)^2)/(2*sigma1*sigma1))*(1-
(p1/indn));
    else if xfeff(i)>1
            ff(i)=exp(-((xf(i)-meant)^2)/(2*sigma2*sigma2))*(1-
(p2/indn));
    else ff(i)=fitintp;
    end
    end
end
fitf=ff;


%calculation of fitness of the males
for i=1:indn/2
     xmeff(i)=round(xm(i),6);
    if xmeff(i)<1
        fm(i)=exp(-((xm(i)-meano)^2)/(2*sigma1*sigma1))*(1-
(p1/indn));
    else if xmeff(i)>1
            fm(i)=exp(-((xm(i)-meant)^2)/(2*sigma2*sigma2))*(1-
(p2/indn));
    else fm(i)=fitintp;
    end
    end
end
fitm=fm;

end

function[tm]=tcalc(tmcr1,tmcr2,indn,loci,tdel,it)

tmcr=zeros(indn/2,loci);

%calculation of t:
for mm=1:indn/2
for m=1:loci
    if tmcr1(mm,m)==1 && tmcr2(mm,m)==1
```

```
        tmcr(mm,m)=it;
    else if tmcr1(mm,m)==1 && tmcr2(mm,m)==0
            tmcr(mm,m)=1;
    else if tmcr1(mm,m)==0 && tmcr2(mm,m)==1
            tmcr(mm,m)=1;
    else if tmcr1(mm,m)==0 && tmcr2(mm,m)==0
            tmcr(mm,m)=0;
    end
    end
    end
    end
end
end
tm1=tdel.*tmcr;
tm=sum(tm1');


end

function[pf]=pcalc(pfcr1,pfcr2,indn,loci,pdel,ip)

pfcr=zeros(indn/2,loci);

%calculation of p:
for ll=1:indn/2
for l=1:loci
    if pfcr1(ll,l)==1 && pfcr2(ll,l)==1
        pfcr(ll,l)=ip;
    else if pfcr1(ll,l)==1 && pfcr2(ll,l)==0
            pfcr(ll,l)=1;
    else if pfcr1(ll,l)==0 && pfcr2(ll,l)==1
            pfcr(ll,l)=1;
    else if pfcr1(ll,l)==0 && pfcr2(ll,l)==0
            pfcr(ll,l)=0;
    end
    end
    end
    end
end
end
pf1=pdel.*pfcr;
pf=sum(pf1');

end

function[xfemcr1,xfemcr2,xmalcr1,xmalcr2]=xmat(xfcr1,xfcr2,xmcr1,xmc
r2,indn,loci,fmat,mmat);

%first half of the individuals generated are females, second half
males%

%genotype of the chromosome from the female:
for i=1:indn
    f=fmat(i); %f is the female that mates in the ith mating event.
    for j=1:loci
```

```
        r1=rand;
        r2=rand;
        if r1<0.5 && r2>0.00001
            crf1(i,j)=xfcr1(f,j);
        else if r1<0.5 && r2<0.00001
                crf1(i,j)=xfcr2(f,j);
        else if r1>0.5 && r2>0.00001
                crf1(i,j)=xfcr2(f,j);
        else crf1(i,j)=xfcr1(f,j);
        end
        end
    end
    end
end
xfemcr1=crf1(1:indn/2,:);
xmalcr1=crf1(indn/2+1:indn,:);

%genotype of the chromosome from the male:
for i=1:indn
    m=mmat(i); %m is the male that mates in the ith mating event%
    for j=1:loci
        r3=rand;
        r4=rand;
        if r3<0.5 && r4>0.00001
            crf2(i,j)=xmcr1(m,j);
        else if r3<0.5 && r4<0.00001
                crf2(i,j)=xmcr2(m,j);
        else if r3>0.5 && r4>0.00001
                crf2(i,j)=xmcr2(m,j);
        else crf2(i,j)=xmcr1(m,j);
        end
        end
    end
    end
end
xfemcr2=crf2(1:indn/2,:);
xmalcr2=crf2(indn/2+1:indn,:);

end

function[tfemcr1,tfemcr2,tmalcr1,tmalcr2]=tmat(tfcr1,tfcr2,tmcr1,tmc
r2,indn,loci,fmat,mmat);

%first half of the individuals generated are females, second half
males%

%genotype of the chromosome from the female:
for i=1:indn
    f=fmat(i);
    for j=1:loci
        r1=rand;
        r2=rand;
        if r1<0.5 && r2>0.00001
            crf1(i,j)=tfcr1(f,j);
        else if r1<0.5 && r2<0.00001
```

```
                crf1(i,j)=tfcr2(f,j);
        else if r1>0.5 && r2>0.00001
                crf1(i,j)=tfcr2(f,j);
        else crf1(i,j)=tfcr1(f,j);
        end
        end
    end
    end
end
tfemcr1=crf1(1:indn/2,:);
tmalcr1=crf1(indn/2+1:indn,:);

%genotype of the chromosome from the male:
for i=1:indn
    m=mmat(i);
    for j=1:loci
        r3=rand;
        r4=rand;
        if r3<0.5 && r4>0.00001
            crf2(i,j)=tmcr1(m,j);
        else if r3<0.5 && r4<0.00001
                crf2(i,j)=tmcr2(m,j);
        else if r3>0.5 && r4>0.00001
                crf2(i,j)=tmcr2(m,j);
        else crf2(i,j)=tmcr1(m,j);
        end
        end
    end
    end
end
tfemcr2=crf2(1:indn/2,:);
tmalcr2=crf2(indn/2+1:indn,:);

end

function[pfemcr1,pfemcr2,pmalcr1,pmalcr2]=pmat(pfcr1,pfcr2,pmcr1,pmc
r2,indn,loci,fmat,mmat);

%first half of the individuals generated are females, second half
males%

%genotype of the chromosome from the female:
for i=1:indn
    f=fmat(i);
    for j=1:loci
        r1=rand;
        r2=rand;
        if r1<0.5 && r2>0.00001
            crf1(i,j)=pfcr1(f,j);
        else if r1<0.5 && r2<0.00001
                crf1(i,j)=pfcr2(f,j);
        else if r1>0.5 && r2>0.00001
                crf1(i,j)=pfcr2(f,j);
        else crf1(i,j)=pfcr1(f,j);
        end
```

```
        end
    end
    end
end
pfemcr1=crf1(1:indn/2,:);
pmalcr1=crf1(indn/2+1:indn,:);

%genotype of the chromosome from the male:
for i=1:indn
    m=mmat(i);
    for j=1:loci
        r3=rand;
        r4=rand;
        if r3<0.5 && r4>0.00001
            crf2(i,j)=pmcr1(m,j);
        else if r3<0.5 && r4<0.00001
                crf2(i,j)=pmcr2(m,j);
        else if r3>0.5 && r4>0.00001
                crf2(i,j)=pmcr2(m,j);
        else crf2(i,j)=pmcr1(m,j);
        end
        end
    end
    end
end
pfemcr2=crf2(1:indn/2,:);
pmalcr2=crf2(indn/2+1:indn,:);

end
```

Assortative mating

mainfile

```
clc
close all
clear all

ngen=50; %number of generations
avg_asso=zeros(100,50);
dev_asso=zeros(100,50);

sig1=0.1; %width of resource distribution

for i=1:length(sig1)
   sig=sig1(i);
   ij=1;
while ij<=100
    [split(ij),avg_asso(ij,:),dev_asso(ij,:)]=symsp_asso(ngen,sig);
    ij=ij+1;
end
end


function[diff,p_asso,p_asso_dev]=symsp_asso(ngenerations,dev)

nloci=20;  % number of loci that control x,t,p and assortativeness%
nind=1000;  %number of individuals%

alpha=5; %strength of sexual selection
delx=2/(2*nloci); %contribution of each locus towards beak size
delt=0.8/(2*nloci); %contribution of each locus towards investment strategy of
males
delp=0.4/(2*nloci); %contribution of each locus towards choosiness of females
dela=1/(2*nloci); %contribution of each locus towards assortativeness
sump=0.4; %maximum permissible choosiness
%width of resouce distribution
sig1=dev;
sig2=dev;

matm=zeros(nind,1); %array of males that mate%
matf=zeros(nind,1); %array of females that mate%

xtrack=zeros(ngenerations,nind); %beak size of individuals of every generation%
ttrack=zeros(ngenerations,nind/2); %investment strategies of the males of every
generation%
ptrack=zeros(ngenerations,nind/2); %choosiness of the females of every generation%
mtrack=zeros(ngenerations,nind);
ftrack=zeros(ngenerations,nind);

fpcr1=zeros(nind/2,nloci); %first strand of female's gene - p
fpcr2=zeros(nind/2,nloci); %second strand of female's gene - p
mpcr1=zeros(nind/2,nloci); %first strand of male's gene - p
mpcr2=ones(nind/2,nloci); %second strand of male's gene - p

ftcr1=zeros(nind/2,nloci); %first strand of female's gene - t
ftcr2=ones(nind/2,nloci); %second strand of female's gene - t
mtcr1=zeros(nind/2,nloci); %first strand of male's gene - t
mtcr2=zeros(nind/2,nloci); %second strand of male's gene - t

fxcr1=zeros(nind/2,nloci); %first strand of female's gene - x
```

```
fxcr2=ones(nind/2,nloci); %second strand of female's gene - x
mxcr1=zeros(nind/2,nloci); %first strand of male's gene - x
mxcr2=ones(nind/2,nloci); %second strand of male's gene - x

fasscr1=zeros(nind/2,nloci); %first strand of female's gene - assortativess
fasscr2=zeros(nind/2,nloci); %second strand of female's gene - assortativeness
masscr1=zeros(nind/2,nloci); %first strand of male's gene - assortativeness
masscr2=ones(nind/2,nloci); %second strand of male's gene - assortativeness


%creation of normal distributions that indicate disruptive selection%
mean1=0.5;
mean2=1.5;


for i=1:ngenerations

    msmallbs=0; %number of individuals with beak size < 1
    mbigbs=0; %number of individuals with beak size > 1
    ngen(i)=i;

    %calculation of assortativeness of the females
    [fscore,assf]=assocalc(fasscr1,fasscr2,masscr1,masscr2,nind,dela);
    p_asso(i)=mean(fscore);
    p1_ass(i)=mean(assf);
    p_asso_dev(i)=std(fscore);


[ff,fm,xavg,xvar,xtot,np1,np2,fx,mx]=fcalc_ass(fxcr1,fxcr2,nind,mxcr1,mxcr2,nloci,
delx,mean1,mean2,sig1,sig2); %function that calculates beak size and fitness of
the individuals


    avgxx(i)=xavg;
    varxx(i)=xvar;
    fitness=cat(1,ff,fm);
    avgf(i)=mean(fitness);

    femfit=ff;
    recif=femfit.^(-1);
    malfit=fm;

    [fp]=pcalc_ass(fpcr1,fpcr2,nind,nloci,delp); %function that calculates p of
every female%
    avgp(i)=mean(fp);
    % calculation of probability that a female finds a partner
    fcost=(exp(-8*fp.*fp.*fp/(sump)^3)).*femfit';
    sumfcost=sum(fcost);
    fprob1=fcost/sumfcost;

    sumf=0;
    for x=1:nind/2
        sumf=sumf+fprob1(x);
        fprob(x)=sumf;
    end

    [mt]=tcalc_ass(mtcr1,mtcr2,nind,nloci,delt); %function that calculates t of
every male%
    avgt(i)=mean(mt);
```

```
    % calculation of probability that a male escapes predation%
    for jj=1:nind/2
        predprop(jj)=exp(-mt(jj)/(1-mt(jj)));
    end

    summcost=sum(predprop);
    predprob=predprop/summcost;
    summ=0;
    for y=1:nind/2
        summ=summ+predprob(y);
        mprob(y)=summ;
    end

    if i>=1
        xtrack(i,:)=xtot;
        ttrack(i,:)=mt;
        ptrack(i,:)=fp;
    end

    nmatef=0.000*ones(nind/2,1);

    %meeting and mating events%
    for p=1:nind
        r1=rand; %selection of female that mates%
        ii=1;
        while r1>fprob(ii)
            ii=ii+1;
        end
        matf(p)=ii;
        nmatef(ii)=nmatef(ii)+1;

      if fscore(ii)>0
         prb=fscore(ii); %assortativness of the female that is selected for mating
         bsc=fx(ii); %beak size of the female selected for mating
        if bsc<1
        for q=1:nind/2
            if mx(q)<1
            fdep(q)=prb*(1-mt(q))*malfit(q)*mprob(q)/(1-prb); %probability that
the selected female meets males whose beak sizes are similar to its own
            matingp(q)=exp(mt(q)*malfit(q)*fp(ii)*alpha); %probability that the
selected female meets mates with males whose beak sizes are similar to its own
            else
            fdep(q)=(1-prb)*(1-mt(q))*malfit(q)*mprob(q)/prb; %probability that
the selected female meets males whose beak sizes are dissimilar to its own
            matingp(q)=exp(mt(q)*malfit(q)*fp(ii)*alpha); %probability that the
selected female mates with males whose beak sizes are dissimilar to its own
            end
        end
        else if bsc>1
            for q=1:nind/2
            if mx(q)>1
            fdep(q)=prb*(1-mt(q))*malfit(q)*mprob(q)/(1-prb); %probability that
the selected female meets males whose beak sizes are similar to its own
            matingp(q)=exp(mt(q)*malfit(q)*fp(ii)*alpha); %probability that the
selected female meets mates with males whose beak sizes are similar to its own
            else
            fdep(q)=(1-prb)*(1-mt(q))*malfit(q)*mprob(q)/prb; %probability that
the selected female meets males whose beak sizes are dissimilar to its own
```

```
            matingp(q)=exp(mt(q)*malfit(q)*fp(ii)*alpha); %probability that the
selected female mates with males whose beak sizes are dissimilar to its own
            end
            end
        end
        end
     else
         for q=1:nind/2
            fdep(q)=0.0001*(1-mt(q))*malfit(q)*mprob(q); %product of the selected
female's fitness with all the males' fitness"
            matingp(q)=exp(mt(q)*malfit(q)*fp(ii)*alpha); %mating probability of
the selected female with all the males in the population
         end
     end

            sumfdep=sum(fdep);
            fdepp=fdep/sumfdep;
            summatp=sum(matingp);
            probmating=matingp/summatp;

            sumffdep=0;
            summating=0;

            for pp=1:nind/2
              sumffdep=sumffdep+fdepp(pp);
              fitnessprob(pp)=sumffdep;
              summating=summating+probmating(pp);
              matprb(pp)=summating;
            end

        %identification of meeting event, and then the mating event%
        iii=1;
        iiii=1;
        while iiii==1
            r2=rand;
            r3=rand;
         while r2>fitnessprob(iii)
            iii=iii+1;
         end
         ev=iii;
         if r3>matprb(ev)
            iiii=1;
         else
             iiii=0;

         end
        end
        matm(p)=ev;
    end

    for b=1:nind
        indf=matf(b);
        indm=matm(b);
        matfemx(b)=fx(indf);
        matmalx(b)=mx(indm);
    end

    if i>=1
        mtrack(i,:)=matmalx;
```

```
        ftrack(i,:)=matfemx;
    end



    %generation of the genotypes of offspring of next generation

[fempcr1,fempcr2,malpcr1,malpcr2]=pmat_ass(fpcr1,fpcr2,mpcr1,mpcr2,nind,nloci,matf
,matm);

[femtcr1,femtcr2,maltcr1,maltcr2]=tmat_ass(ftcr1,ftcr2,mtcr1,mtcr2,nind,nloci,matf
,matm);

[femxcr1,femxcr2,malxcr1,malxcr2]=xmat_ass(fxcr1,fxcr2,mxcr1,mxcr2,nind,nloci,matf
,matm);

[asscr1_f,asscr2_f,asscr1_m,asscr2_m]=assmat(fasscr1,fasscr2,masscr1,masscr2,nind,
nloci,matf,matm);

    %reassignment of female and male genotypes%
    fpcr1=fempcr1;
    fpcr2=fempcr2;
    mpcr1=malpcr1;
    mpcr2=malpcr2;

    ftcr1=femtcr1;
    ftcr2=femtcr2;
    mtcr1=maltcr1;
    mtcr2=maltcr2;

    fxcr1=femxcr1;
    fxcr2=femxcr2;
    mxcr1=malxcr1;
    mxcr2=malxcr2;

    fasscr1=asscr1_f;
    fasscr2=asscr2_f;
    masscr1=asscr1_m;
    masscr2=asscr2_m;
end

femxf=ftrack(ngenerations-1,:);
malxf=mtrack(ngenerations-1,:);
matxf=[femxf malxf];

%calculation of intensity of split
diff=1;
bs1=0;
for pp=1:(2*nind)
    if matxf(pp)==1
        bs1=bs1+1;
    else
        bs1=bs1+0;
    end
end


if bs1~=0
    diff=0;
```

```
else
nsmallx=0;
nbigx=0;
 for hh=1:(nind*2)
    if matxf(hh)<1
        nsmallx=nsmallx+1;
    else if matxf(hh)>1
            nbigx=nbigx+1;
    end
    end
 end

  if nsmallx==0 && nbigx~=0
    diff=-1;
  else if nsmallx~=0 && nbigx==0
          diff=-1;
  else if nsmallx<2 || nbigx<2
          diff=-1;
  else if (nsmallx~=0 && nbigx~=0) && (nsmallx>2 && nbigx>2)

     smallx=zeros(nsmallx,1);
     bigx=zeros(nbigx,1);

     fmatx=sort(matxf);

     smallx=(fmatx(:,1:nsmallx))';
     bigx=(fmatx(:,(nsmallx+1):end))';

     pd1=fitdist(smallx,'Normal');
     m1=mean(pd1);
     dev1=std(pd1);
     pd2=fitdist(bigx,'Normal');
     m2=mean(pd2);
     dev2=std(pd2);

     diff=(m2-m1)/(dev2+dev1);
  end
  end
  end
  end
end
end

function[totalf,scoref]=assocalc(cr1assf,cr2assf,cr1assm,cr2assm,indn,adel)

scoref=0;
scorem=0;
femasscr=cr1assf+cr2assf;
malasscr=cr2assm+cr1assm;

totalf=adel*sum(femasscr'); %assortativeness of the females


for i=1:indn/2
    if totalf(i)>0
        scoref=scoref+1;
    end
end
```

```
end
```
